# Percutaneous and surgical management of aortic stenosis in the SWEDEHEART registry (2013–2023): a nationwide observational study

**DOI:** 10.1016/j.lanepe.2025.101520

**Published:** 2025-11-03

**Authors:** Johannes Tödt, Sasha Koul, Troels Yndigegn, Oskar Angerås, Henrik Bjursten, Shahab Nozohoor, Örjan Friberg, Jenny Backes, Stefan James, Björn Redfors, Patrik Gilje, Ashkan Labaf, Henrik Hagström, Andreas Rück, Matthias Götberg, Moman Aladdin Mohammad

**Affiliations:** a Division of Cardiology, Department of Clinical Sciences Lund, Lund University, Lund, Sweden; b Department of Cardiology, Skåne University Hospital, Lund, Sweden; c Department of Thoracic Surgery and Cardiology, Sahlgrenska University Hospital, Gothenburg, Sweden; d Department of Molecular and Clinical Medicine, Institute of Medicine, Gothenburg University, Gothenburg, Sweden; e Department of Cardiothoracic Surgery, Anesthesia and Intensive Care, Lund University/Skåne University Hospital, Lund, Sweden; f School of Medica Sciences, Faculty of Medicine and Health, Örebro University, Örebro, Sweden; g Department of Cardiothoracic and Vascular Surgery, Örebro University Hospital, Örebro, Sweden; h Department of Medical Sciences, Uppsala University, Uppsala, Sweden; i Division of Cardiology, Department of Medicine, Karolinska Institutet, Stockholm, Sweden; j Department of Public Health and Clinical Medicine, Umeå University, Umeå, Sweden

**Keywords:** Transcatheter aortic valve implantation (TAVI), Surgical aortic valve replacement (SAVR), Temporal trends, Aortic stenosis, SWEDEHEART, Valve replacement outcomes, Aortic valve implantation, Prosthetic aortic valve implantation, SWENTRY, SCSR

## Abstract

**Background:**

Management of severe aortic stenosis (AS) has evolved over the past decade, driven by the widespread adoption of transcatheter aortic valve implantation (TAVI). This study aims to assess trends in procedural volumes, patient characteristics, and outcomes for patients undergoing TAVI or surgical aortic valve replacement (SAVR) in Sweden.

**Methods:**

This was a descriptive, non-comparative, nationwide cohort study using the SWEDEHEART registry. We included 21,383 patients who underwent TAVI or SAVR between 2013 and 2023 (11,366 TAVI and 10,017 SAVR). Trends in patient characteristics, preoperative risk, complications and mortality were examined.

**Findings:**

TAVI procedures increased from 307 (26.1%, n = 307/1174) in 2013 to 1851 (71.2%, n = 1851/2601) in 2023, while SAVR volumes declined from ∼1000 annually before 2018 to roughly 750 procedures annually. Median age of TAVI patients were 81 (IQR 77, 85) years and 71 (IQR 65, 76) years for SAVR patients. The median EuroSCORE II for TAVI decreased from 5.6 (IQR 3.3, 10.2) to 2.7 (IQR 1.7, 4.6) (p = 0.002), and STS-PROM from 3.3 (IQR 1.9, 4.1) to 1.6 (IQR 1.1, 2.8) (p = 0.0021). Among SAVR patients, EuroSCORE II decreased from 1.5 (IQR 1.0, 2.3) to 1.3 (IQR 0.9, 2.1) (p = 0.022) and STS-PROM from 1.8 (IQR 1.2, 3.0) to 1.6 (IQR 1.1, 2.6) (p = 0.0082). Any in-hospital complications declined significantly for TAVI (29.2%, n = 210/719 to 13.2%, n = 244/1851), while SAVR complication rates increased slightly (18.4%, n = 354/1921 to 18.7%, n = 140/750). In-hospital mortality for TAVI declined from 3.6% (n = 26/719) to 1.0% (n = 18/1851), and 1-year mortality from 11.1% to 6.9% (p = 0.019). SAVR in-hospital all-cause death decreased from 1.6% to 0.4% (n = 3/750) and 5.0% to 2.2% for 1-year mortality (p = 0.013).

**Interpretation:**

TAVI has become the predominant treatment strategy for AS in Sweden expanding access within the treated cohort. Despite this, current 2023 SAVR results demonstrate similar in-hospital complication rates compared to TAVI (18.7% vs 13.2%), but lower in-hospital (0.4% vs 1.0%) and 1-year mortality rates (2.2% vs 6.9%).

**Funding:**

This study was supported by ALF and national research funding bodies.

**Research in context**

**Evidence before this study**

We searched PubMed for studies published from database inception to September 1, 2025, using the terms “aortic stenosis”, “transcatheter aortic valve implantation”, “TAVI”, “TAVR”, “surgical aortic valve replacement”, “SAVR”, “temporal trends”, “outcomes”. No language or date restrictions were applied. Randomised trials show comparable early-, mid- and long-term outcomes for TAVI and SAVR. Some registry analysis suggests divergence at longer follow-up. Registries report rising TAVI use and improving results, but most examine either TAVI or SAVR alone, cover shorter periods or lack discussion regarding TAVIs implication on SAVR trends.

**Added value of this study**

Using the mandatory nationwide SWEDEHEART registry, we describe both TAVI and SAVR in Sweden from 2013 to 2023 (n = 21,383). To our knowledge, this is the first nationwide study to examine TAVI and SAVR together, assessing how the rise of TAVI has influenced the use, patient profiles and outcomes of SAVR over time. We present temporal trends in procedural volumes, patient characteristics, preoperative risk, valve morphology, access strategy and outcomes across both TAVI and SAVR, including subgroups with concomitant coronary revascularization (TAVI + PCI and SAVR + CABG). Our results show a marked increase in TAVI use, stable SAVR volumes, declining preoperative risk (EuroSCORE II and STS-PROM), and improved in-hospital and 1–5-year survival across both modalities. The study design is descriptive and not intended to compare modalities.

**Implications of all the available evidence**

Contemporary care is shifting towards TAVI while SAVR remains essential for patients with longer life expectancy, complex anatomy and patients requiring multiple anatomical interventions. Improving risk profiles and outcomes across both strategies support heart-team, anatomy-led selection and lifetime management planning. Future research should extend to encompass the effect of coronary revascularization and its timing, lifelong management of possible valve-in-valve procedures and continuous evaluation of how TAVI impacts the field of aortic stenosis.

## Introduction

Aortic stenosis (AS) is the most common valvular heart disease in the Western world, affecting 2–7% of individuals over 65 years old, with prevalence rising due to an ageing population.^1^^,^^2^ Severe symptomatic AS, if untreated, has a poor prognosis, with a 4-year mortality rate exceeding 60%.^3^ While SAVR has traditionally been the standard treatment, TAVI, initially developed for patients with high surgical risk has expanded to include intermediate- and low-risk groups. European guidelines recommend TAVI primarily for patients with suitable anatomy over 75 years or who are at increased surgical risk, while American guidelines extend its use to those 65 years and older across all risk categories. Randomised trials have shown comparable outcomes between TAVI and SAVR through mid-term and long-term follow-up although recent meta-analyses suggest potential late differences that may favour SAVR in some endpoints beyond 2 years follow-up.^4^ Meanwhile, lifetime outcomes in younger patients with longer life expectancy remain an area of active study.

Temporal trends indicate increasing TAVI use in lower-risk populations, with significant geographic variations. In Europe, registry data show a gradual shift toward younger TAVI patients, while U.S. studies report that 40–55% of prosthetic aortic valve implantation recipients under 65 years now undergo TAVI.^5^, ^6^, ^7^, ^8^, ^9^, ^10^, ^11^, ^12^ While emerging data suggest encouraging long-term valve durability in low-risk patients, it remains unclear whether these outcomes translate equivalently in younger individuals who face a higher cumulative lifetime risk of valve degeneration and reintervention. Coronary access is a main concern as indications expand to younger patients with longer life expectancy, where future reinterventions such as TAVI-in-TAVI or TAVI-in-SAVR may become increasingly prevalent. Despite numerous reports on TAVI's growth, few have examined how its expansion has impacted SAVR, which remains essential for younger patients, those with more anatomically complex valve disease, or those requiring additional procedures like CABG. Monitoring TAVI's increasing use and its impact on patient selection is crucial, as these shifts influence the characteristics and outcomes of prosthetic aortic valve implantation patients over time.

This study utilizes SWEDEHEART registry data to evaluate real-world trends in the management of AS in Sweden from 2013 to 2023, assessing procedural volumes, patient demographics, risk profiles, and clinical outcomes to provide insight into evolving treatment strategies and guideline adherence (Table 1).

**Table 1:** Baseline characteristics.

Intervention	TAVI	SAVR	Total
N = #	11,366	10,017	21,383
Age	81 (77, 85)	71 (65, 76)	77 (70, 82)
Sex			
Female	5216 (45.9%)	3347 (33.4%)	8563 (40.0%)
Male	6150 (54.1%)	6670 (66.6%)	12,820 (60.0%)
Hypertension	8690 (76.9%)	4716 (47.1%)	13,406 (62.9%)
Missing	0 (0.0%)	3475 (34.1%)	3475 (16.3%)
Diabetes	2895 (25.6%)	2334 (23.3%)	5229 (24.6%)
Atrial fibrillation	4042 (35.8%)	1604 (17.8%)	5646 (27.8%)
Previous PCI	1998 (17.6%)	616 (6.1%)	2614 (12.2%)
Chronic pulmonary disease	1826 (16.2%)	838 (8.4%)	2664 (12.5%)
Peripheral vessel disease	1668 (14.8%)	506 (5.1%)	2174 (10.2%)
Previous cardiac surgery	1723 (15.2%)	240 (2.4%)	1963 (9.2%)
Recent MI	413 (3.7%)	697 (7.0%)	1110 (5.2%)
Pulmonary hypertension			
0–30 mmHg	2149 (24.5%)	7957 (80.7%)	10,106 (54.2%)
31–55 mmHg	5529 (63.0%)	1377 (14.0%)	6906 (37.1%)
56–120 mmHg	1096 (12.5%)	215 (2.2%)	1311 (7.0%)
NYHA Class			
Class I	261 (2.3%)	793 (7.9%)	1054 (5.0%)
Class II	2408 (21.3%)	4322 (43.3%)	6730 (31.6%)
Class III	7424 (65.7%)	4475 (44.8%)	11,899 (55.9%)
Class IV	1192 (10.6%)	315 (3.2%)	507 (7.1%)
EuroSCORE II	3.6 (2.1, 6.3)	1.4 (1.0, 2.2)	2.2 (1.3, 4.3)
STS-PROM	1.9 (1.2, 3.3)	1.7 (1.1, 2.8)	1.8 (1.1, 3.0)
Left ventricular ejection fraction			
LVEF >50%	7985 (71.0%)	7859 (78.6%)	15,844 (74.6%)
LVEF 31–50%	1916 (17.0%)	1707 (17.1%)	3623 (17.1%)
LVEF 21–30%	934 (8.3%)	302 (3.0%)	1236 (5.8%)
LVEF 20% or less	410 (3.6%)	119 (1.2%)	529 (2.5%)
Bicuspid/tricuspid valve			
Bicuspid	860 (7.6%)	1342 (13.4%)	2202 (10.3%)
Tricuspid	9067 (79.8%)	2538 (25.3%)	11,605 (54.3%)
Other	0 (0.0%)	52 (0.5%)	52 (0.2%)
Missing	1439 (12.7%)	6085 (607%)	7524 (35.2%)

## Methods

This study utilized data from the SWEDEHEART registry, a nationwide quality of cardiac care umbrella registry including several sub-registries such as the Swedish Transcatheter Cardiac Intervention Registry (SWENTRY) which enrols all patients undergoing TAVI in eight centres in Sweden and the Swedish Cardiac Surgery Registry (SCSR) which documents data on all patients undergoing any cardiac surgical procedure. All TAVI centres have available thoracic surgery in case of bail-out thoracic procedures and all patients planned for intervention undergo a mandatory multidisciplinary heart-team review before allocation to either strategy. All eight Swedish TAVI centres operate within networks with on-site or immediate surgical backup, and all cardiac surgery centres contribute to SCSR. The SWEDEHEART registry has shown over 95% accuracy with regards to follow-up and patient characteristics.^13^ Baseline characteristics, including age, sex, comorbidities, echocardiographic data, New York Heart Association (NYHA) functional class, left ventricular ejection fraction (LVEF), measures of aortic stenosis severity and angiographic findings, were collected from respective registries. Preoperative risk scores (EuroSCORE II and STS-PROM) were calculated for all patients using online available coefficients and calculator models.^14^^,^^15^ Registries were linked to the Swedish Coronary Angiographic and Angioplasty Registry (SCAAR) to obtain data on coronary angiographies and percutaneous coronary interventions. In addition, deterministic linkage using a unique personal identifier for Swedish inhabitants to the National Population Registry allowed for the collection of data relating to vital status.

### Study design

This was a descriptive, non-comparative, nationwide cohort study designed to characterise temporal trends within each modality (TAVI and SAVR) from 2013 to 2023. Hence, we did not construct cross-modality adjusted models (e.g., Cox or multivariable regression contrasting TAVI vs SAVR). We included all patients who underwent TAVI or SAVR between 2013 and 2023. Patients were included if they had severe aortic stenosis as the main indication for intervention. Patients without a Swedish personal identification number were excluded for follow-up reasons. All procedures analysed were first-time aortic valve interventions. Patients with more than one intervention were included with their first intervention as basis for baseline characteristics and complications across the study period. If patients underwent both TAVI and SAVR on the same day, it was assumed that SAVR served as a bail-out procedure, with TAVI considered the index intervention. Patients undergoing SAVR combined with or without additional surgical coronary artery bypass grafting (CABG) were included. Patients undergoing SAVR with other valvular interventions such as mitral and pulmonary valve surgery were excluded. In addition, patients with aortic disease and active endocarditis were excluded in the aortic surgery cohort. Inclusion and exclusion criteria and patients included and excluded are visualised in Supplementary Figure 1. Patients were stratified into risk categories based on EuroSCORE II or STS-PROM: low risk (<4%), intermediate risk (4–8%), and high risk (>8%). The definition of other major concomitant surgeries that were included as well as excluded is summarised in Supplementary Table 1. Patients who underwent TAVI with percutaneous coronary intervention (PCI) were included if PCI was performed within 90 days of the TAVI procedure. Temporal trends in procedural volumes and patient profiles were analysed over the study period.

### Ethics approval

This study was conducted in accordance with the Declaration of Helsinki, and ethical approval was obtained from the Swedish ethical review authority (approval number: DNR 2023-00201-01). The requirement for individual informed consent was waived because the study utilized de-identified data from the national SWEDEHEART registry.

### Clinical outcomes

Temporal trends in in-hospital mortality as well as in-hospital complications: stroke, myocardial infarction, major bleeding, and permanent pacemaker implantation are routinely collected in the registry and were assessed in this study. The reporting of pacemaker implantation changed over the study period, leading to potential variability in outcomes for both TAVI and SAVR. Prior to 2017, pacemaker implantation in the TAVI group was recorded as the presence of a pacemaker at discharge. After 2017, this definition was updated to capture the acquisition of a new pacemaker during the hospital stay. For the SAVR group, the recording of pacemaker implantation began in 2017, limiting the availability of earlier data for comparison. Outcome definitions are detailed in Supplementary Table 2. Temporal trends in all-cause mortality were assessed at 30 days as well as 1, 3 and 5 years.

### Statistical analysis

Continuous variables were summarized using medians and interquartile ranges (IQRs) and between-group comparisons of continuous variables were performed using the Mann–Whitney U test, a non-parametric method suitable for non-normally distributed data. Categorical variables were presented as frequencies with corresponding percentages, and group differences were assessed using Pearson's chi-square (χ^2^) test. Temporal trends were assessed using descriptive statistics and statistically assessed using the non-parametric linear-by-linear Mantel–Haenszel test. All-cause death at 1-, 3-, and 5-years post-procedure was estimated using Kaplan–Meier survival analysis with start at procedure date and censor at death or last follow-up. Event rates were derived accordingly, and differences in temporal mortality trends were evaluated using non-parametric tests for trend across ordered groups. Multivariable Cox regression models adjusted for EuroSCORE II and sex were used in Supplementary analysis. All statistical analyses were conducted using Stata Statistical Software: Release 18 (StataCorp LLC, College Station, TX, USA). A two-sided p-value of <0.05 was considered indicative of statistical significance throughout all analyses.

### Role of funding sources

The funding agencies were not involved in the study design, collection of data, analysis of data, interpretation of data, writing of the manuscript, approving the manuscript or in the decision to submit manuscript for publication. The decision to submit manuscript was solely the authors.

## Results

### Temporal trends in procedural volumes and patient characteristics

**Fig. 1:**
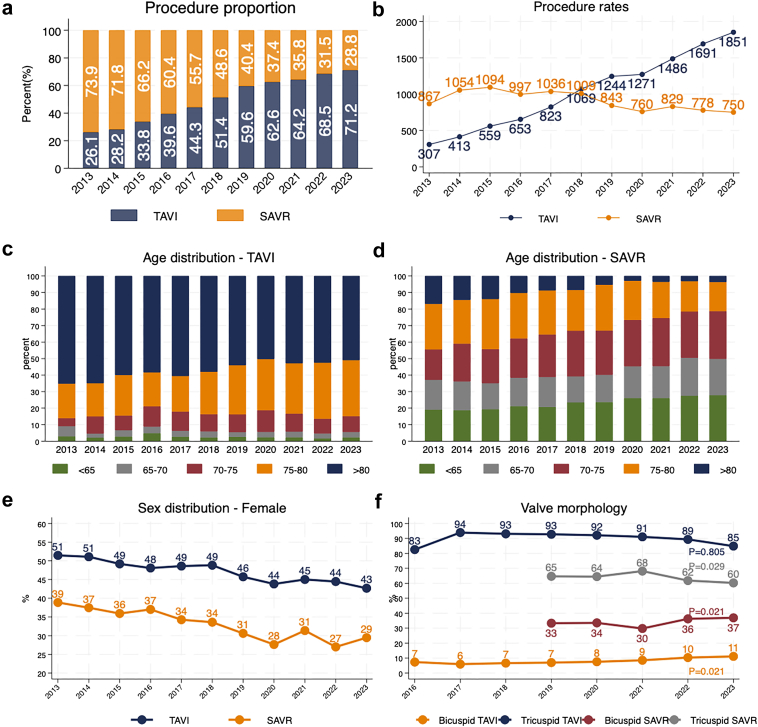
**Temporal trends in prosthetic aortic valve implantation procedures by type, age, and sex, 2013–2023.** (a) Proportion of TAVI and SAVR procedures over time, expressed as percentages of all AVR procedures. (b) Annual procedural volumes for TAVI and SAVR, expressed as the number of procedures performed per year. (c) Age distribution of patients undergoing TAVI, grouped by age categories (<65, 65–70, 70–75, 75–80, and >80 years). (d) Age distribution of patients undergoing SAVR, grouped by the same age categories. (e) Proportion of female patients undergoing TAVI and SAVR over time. (f) Proportion of different valve morphology of TAVI and SAVR.

A total of 21,383 patients who underwent prosthetic aortic valve implantation between 2013 and 2023 were included: 11,366 TAVI and 10,017 SAVR patients. TAVI volumes increased from 307 procedures in 2013 to 1851 in 2023, rising from 26.1% (n = 307/1174) to 71.2% (n = 1851/2601) of all prosthetic aortic valve implantation procedures, Fig. 1a, b. TAVI + PCI cases grew from 81 in 2013 to 125 in 2023, but their proportion declined from 11.3% (n = 81/719) to 6.8% (n = 125/1851) (Supplementary Table 3) (p = 0.018). SAVR volumes remained stable, with a slight decline from 867 in 2013 and reaching a peak of 1094 procedures in 2015 to 750 in 2023 (Fig. 1b). SAVR + CABG decreased from 34.9% (n = 670/1921) to 31.1% (n = 233/750) of SAVR procedures (p = 0.095). The proportion of patients with tricuspid valve morphology undergoing TAVI remained relatively stable over time ranging from 83% (n = 539/653) in 2016 to 85% (n = 1571/1851) in 2023, (p = 0.806). Conversely, the proportion of tricuspid SAVR patients initially increased from 65% (n = 545/843) in 2019 and peaking at 68% (n = 565/829) in 2021, followed by a decline to 60% (n = 452/750) in 2023 (p = 0.029). Meanwhile, bicuspid SAVR increased from 33% (n = 281/843) in 2019 to 37% (n = 277/750) in 2023, (p = 0.021). Bicuspid TAVI also showed a gradual increase from 7% (48/653) in 2016 to 11% (206/1851) in 2023, (p = 0.021) (Fig. 1f). The proportion of mechanical valve prosthesis in the SAVR cohort increased as the frequency of mechanical SAVR (M-SAVR) increased while biological SAVR decreased over time (Supplementary Figure 2b). Transfemoral access for TAVI was the preferred strategy during the study period, increasing from 87.7% (630/719) in 2013–2014 to 98% (n = 1814/1851) in 2023 (p < 0.0001) (Supplementary Table 3). The median age of TAVI patients decreased from 84 (IQR 78, 87) years in 2013 to 81 (IQR 77, 84) years in 2023 (p < 0.0001), while SAVR patients median age declined from 73 (IQR 66, 79) to 70 (IQR 64, 74) years (p < 0.0001). The proportion of women decreased in both groups, from 51% (n = 158/307) to 43% (n = 790/1851 in the TAVI cohort and from 39% (n = 337/867) to 29% (n = 221/750) in the SAVR cohort (TAVI: p < 0.0001; SAVR: p < 0.0001). Prevalence of chronic pulmonary disease declined from 18.5% (n = 133/719) to 13.2% (n = 235/1851) in TAVI (p < 0.0001) and from 9.7% (n = 185/1921) to 8.4% (n = 63/750) in SAVR (p < 0.0001). Prevalence of peripheral vessel disease decreased from 16.1% (n = 115/719) to 11.8% (n = 211/1851) in TAVI (p < 0.0001) and from 6.7% (n = 127/1921) to 3.7% (n = 28/750) in SAVR (p < 0.0001). Prevalence of diabetes among patients referred for SAVR increased significantly over time (p < 0.0001) but remained stable in TAVI. Prior cardiac surgery in TAVI patients decreased from 24.1% (n = 143/719) to 10.6% (n = 196/1851) (p < 0.0001). The proportion of TAVI patients with preserved LVEF, defined as LVEF more than 50%, increased from 60.4% (n = 434/719) to 76.3% (n = 1347/1851) (p < 0.0001), while proportion of patients with severely reduced LVEF, defined as LVEF under 20%, decreased from 9.6% (n = 69/719) to 1.4% (n = 24/1851). In the SAVR cohort the proportion with preserved LVEF increased from 76.4% (n = 1459/1921) to 79.7% (n = 598/750), while patients with severely reduced LVEF increased from 1.1% (n = 21/1921) to 2.0% (n = 15/750). The proportion of patients undergoing concomitantly revascularization with LVEF <20% differed between the two modalities. For TAVI the proportion ranged from 1.5% (n = 11/719) to 0.2% (n = 5/1851) and for SAVR from 0.4% (n = 8/1921) to 1.2% (n = 9/750). The proportion of TAVI patients with mild pulmonary hypertension (0–30 mmHg) increased from 18.1% (n = 114/719) to 32.0% (n = 420/1851), while severe pulmonary hypertension (56–120 mmHg) decreased from 18.9% (n = 119/719) to 8.6% (n = 113/1851) (p < 0.0001). Prevalence of mild pulmonary hypertension (0–30 mmHg) decreased in SAVR patients from 82.6% (n = 1462/1921) to 78.0% (n = 585/750) (p < 0.0001) and prevalence of severe pulmonary hypertension (56–120 mmHg) decreased from 2.7% (n = 48/1921) to 1.3% (n = 10/750) (p < 0.0001). NYHA functional class improvements were observed in both groups (Supplementary Tables 2 and 3).

### The shift in surgical risk over time

**Fig. 2:**
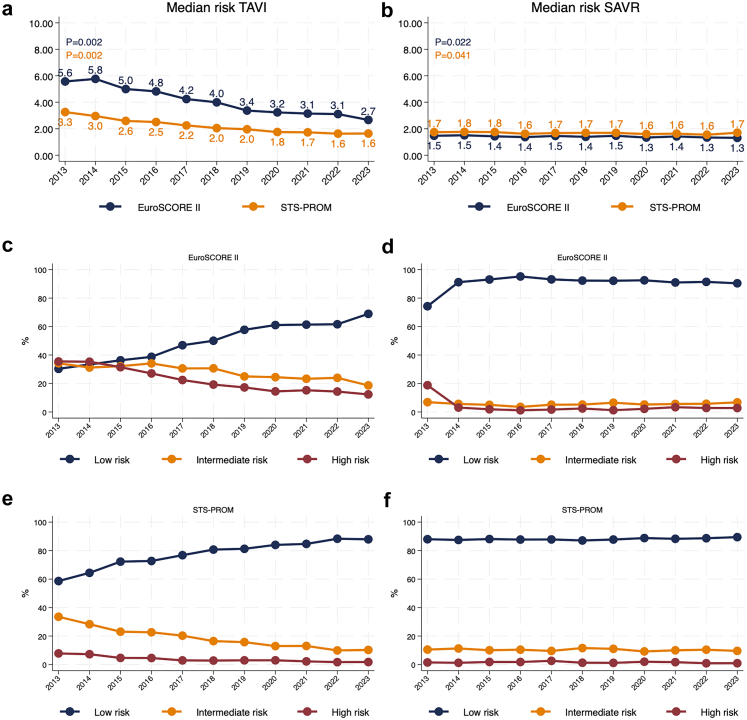
**Temporal trends in surgical risk profiles using EuroSCORE II and STS-PROM for TAVI and SAVR patients, with distribution across risk categories.** (a, b) Median pre-procedural surgical risk, measured using EuroSCORE II and STS-PROM, for TAVI (a) and SAVR (b) patients from 2013 to 2023. (c–f) Distribution of patients by surgical risk categories for EuroSCORE II (c, d) and STS-PROM (e, f), stratified as low risk (0–4), intermediate risk (4–8), and high risk (>8).

**Fig. 3:**
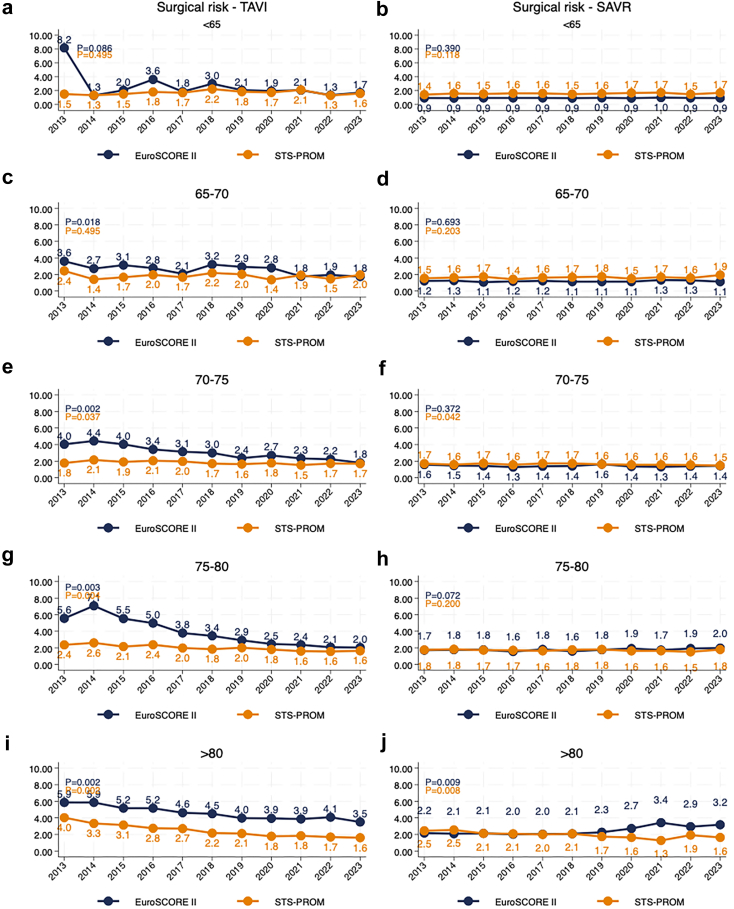
**Temporal trends in EuroSCORE II and STS-PROM stratified by age group for TAVI and SAVR patients (2013**–**2023).** (a,c,e,g,i) Surgical risk scores over time, measured using EuroSCORE II and STS-PROM, for TAVI patients stratified by age groups: <65 years (a), 65–70 years (c), 70–75 years (e), 75–80 years (g), and >80 years (i). (b,d,f,h,j) Surgical risk scores over time, measured using EuroSCORE II and STS-PROM, for SAVR patients stratified by the same age groups: <65 years (b), 65–70 years (d), 70–75 years (f), 75–80 years (h), and >80 years (j).

Fig. 2 illustrates temporal changes in surgical risk scores. In the TAVI group, median EuroSCORE II declined from 5.6 (IQR 3.3, 10.2) to 2.7 (IQR 1.7, 4.6) (p = 0.0021), and STS-PROM from 3.3 (IQR 1.9, 4.1) to 1.6 (IQR 1.1, 2.8) (p = 0.0022). SAVR patients showed smaller decline: EuroSCORE II decreased from 1.5 (IQR 1.0, 2.3) to 1.3 (IQR 0.9, 2.1) (p = 0.022) and STS-PROM from 1.8 (IQR 1.2, 3.0) to 1.6 (IQR 1.1, 2.6) (p = 0.019). The proportion of low-risk TAVI patients, EuroSCORE II (<4%) increased, from 30.3% (n = 93/307) to 68.9% (n = 1276/1851) (p = 0.019), and STS-PROM (<4%) from 58.6% (n = 180/307) to 88.0% (n = 1628/1851) (p = 0.0023). In contrast, the proportion of low-risk SAVR patients remained stable. The largest reductions in TAVI risk scores occurred among older patients: in those aged 75–80 years, EuroSCORE II decreased from 5.6 to 2.0 (p = 0.0031) and STS-PROM from 2.4 to 1.6 (p = 0.032). In patients over 80 years, EuroSCORE II decreased from 5.9 to 3.5 (p = 0.0024), and STS-PROM from 4.0 to 1.6 (p = 0.0022). Fig. 3 shows in detail trends in median EuroSCORE II and STS-PROM stratified by five different age groups and TAVI and SAVR.

Risk scores in the SAVR cohort remained generally stable over time in younger patients. However, in older age groups, a slight increase in risk scores was observed. Among patients 80 years or older, STS-PROM and EuroSCORE II showed diverging trends with STS-PROM declining from 2.5 to 1.6 (p = 0.0082) and EuroSCORE II increasing from 2.2 to 3.2 (p = 0.0091).

### Temporal trends in complications and mortality

**Fig. 4:**
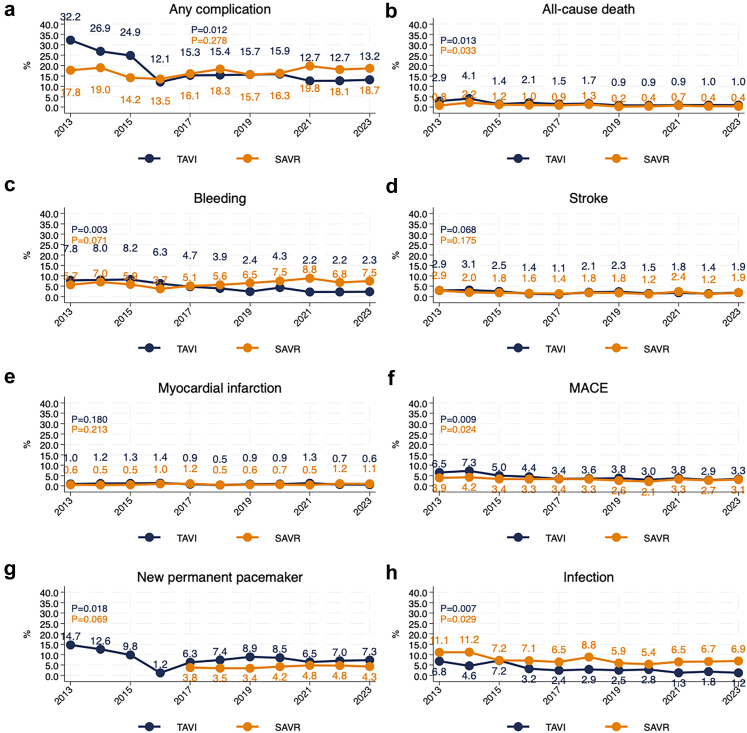
**Temporal trends in in-hospital complications for TAVI and SAVR patients (2013**–**2023)**. (a) Proportion of patients experiencing any in-hospital complication over time. (b) Proportion of patients experiencing major adverse cardiac events (MACE) during hospitalization. (c) In-hospital all-cause mortality rates over time. (d) Rates of bleeding complications during hospitalization. (e) Incidence of stroke during hospitalization. (f) Incidence of myocardial infarction during hospitalization. (g) Rates of pacemaker implantation following the procedure. (h) Incidence of infections during hospitalization.

**Fig. 5:**
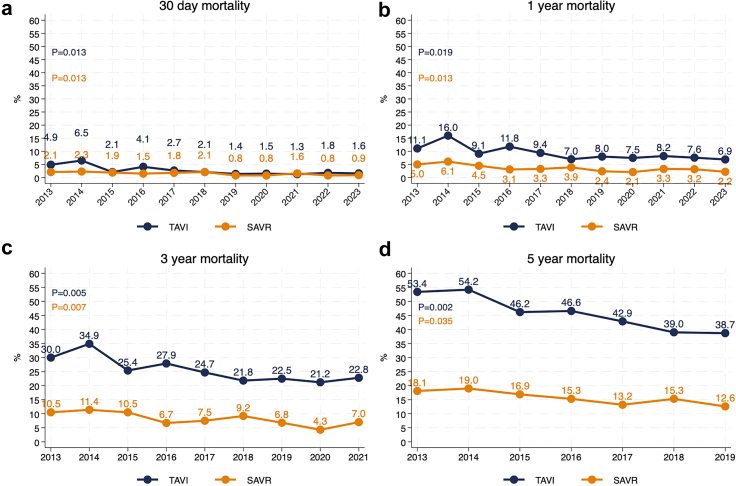
**Temporal trends in all-cause mortality at 30 days, 1, 3, and 5 years for TAVI and SAVR patients (2013**–**2023)**. (a) 30-day all-cause mortality rates over time for TAVI and SAVR (b) One-year all-cause mortality rates over time for TAVI and SAVR patients. (c) Three-year all-cause mortality rates over time for TAVI and SAVR patients. (d) Five-year all-cause mortality rates over time for TAVI and SAVR patients.

In-hospital major adverse cardiac events (MACE) in the TAVI cohort declined from 6.5% (n = 20/307) to 3.3% (n = 62/1851) (p = 0.0092). Major bleeding rates decreased from 7.8% (24/307) in 2013 to 2.3% (n = 43/1851) in 2023 (p = 0.003), and in-hospital mortality from 2.9% (n = 9/307) to 1.0% (n = 18/1851) (p = 0.013). Stroke rates remained stable, ranging from 2.9% (n = 9/307) to 1.9% (n = 35/1851) (p = 0.068). Pacemaker implantation showed a significant reduction over time, decreasing from 14.7% (n = 45/307) in 2013 to 7.3% (n = 136/1851) in 2023 (p = 0.018). Any in-hospital complication rate declined significantly from 32.2% (n = 99/307) to 13.2% (n = 244/1851) (p = 0.012) (Fig. 4). 30-day and 1-year all-cause mortality declined from 4.9% to 1.6% and 11.1% to 6.9% respectively (p = 0.013 and p = 0.019). Three-year and 5-year all-cause mortality declined, from 30.0% to 22.8% (p = 0.0052) and 53.4% to 38.7% (p = 0.0021), respectively (Fig. 5).

For SAVR in hospital MACE decreased from 3.9% (n = 34/1921) to 3.1% (n = 23/750) (p = 0.024). Major bleeding rates increased from 5.7% (n = 49/1921) to 7.5% (n = 56/750) (p = 0.071), and stroke rates showed no significant change, remaining between 2.9% (n = 25/1921) and 1.9% (n = 14/750) (p = 0.18). Pacemaker implantation recorded from 2017 and onward ranged from 3.8% (n = 39/1036) to 4.3% (n = 32/750) (p = 0.069). In-hospital mortality decreased from 0.8% (n = 7/1921) in 2013 to 0.4% (n = 3/750) in 2023 (p = 0.033) (Fig. 4). Any in-hospital complication rates were stable, ranging from 17.8% (n = 154/1921) to 18.7% (n = 140/750) (p = 0.28). 30-day and 1-year mortality declined from 2.1% to 0.9% and 5.0% to 2.2% (p = 0.012, p = 0.013 respectively). Three-year and 5-year all-cause mortality declined significantly, from 10.5% to 7.0% (p = 0.0071) and 18.1% to 12.6% (p = 0.0072, p = 0.035 respectively) (Fig. 4).

In additional analyses, complication trends stratified by concomitant revascularization demonstrated higher complication rates in SAVR + CABG patients than patients undergoing isolated SAVR (Supplementary Figure 3), while TAVI + PCI patients showed similar outcomes as isolated TAVI (Supplementary Figure 4). Looking at 1-year mortality stratified by modality and concomitant coronary revascularization, unadjusted crude cumulative risk was lowest for isolated SAVR and highest with TAVI + PCI (Supplementary Figure 10). Risk-stratified analyses revealed significantly improved survival among intermediate-risk TAVI patients at all follow-up intervals, whereas no improvement was observed in high-risk patients, and only short-term benefit was seen in low-risk patients over time (Supplementary Figure 5). For SAVR, mortality remained consistently low across risk groups with limited improvement over time, except for intermediate-risk patients at 1- and 3-year follow-up (Supplementary Figure 6). Ten-year mortality for TAVI was 83% and for SAVR 42%. However, only 0.5% of TAVI patients and 8% of SAVR patients remained at risk at 10 years due to censoring and loss to follow-up (Supplementary Figure 7). Stratification by LVEF demonstrated a stepwise increase in 1-year mortality with lower LVEF in both TAVI and SAVR patients (Supplementary Figures 8 and 9). Finally, a cox regression model adjusted for EuroSCORE II and sex showed no consistent improvement in 30-day nor 1-year mortality for SAVR over time (Supplementary Tables 8 and 11), whereas TAVI patients demonstrated a general reduction in later years (Supplementary Tables 9 and 10).

## Discussion

This Swedish nationwide study describes the transformation of aortic valve intervention over the past decade. Our key finding is that in the SWEDEHEART prosthetic aortic valve implantation cohort, TAVI accounted for an increasing share of procedures (26.1–71.2%), while SAVR volumes were stable initially and fell modestly after 2018. Additionally, periprocedural complications and outcomes for both TAVI and SAVR have continuously improved during the study period. These findings provide valuable insights into the evolution of prosthetic aortic valve implantation practices, can be used to inform patient consent and highlight areas for further innovation and refinement in the management of severe aortic stenosis.

The SWENTRY registry represents a nationwide cohort from a free-for-all healthcare system that includes all-comer patients. Like previous temporal trends studies, we observed a significant rise in TAVI procedures with simultaneously improved outcome.

TAVI data from US Medicare (2012–2019) showed a decline in 30-day mortality from 6.3% to 2.0% which aligns with the results in our study with a decrease from 4.9% to 1.6%.^16^ Similarly, the German TAVI registry (2013–2020) showed low in-hospital mortality (1.6%) and stroke rates (3.0%) but higher pacemaker implantation rates (13.8%) which was 7.4% in our study.^6^ The Danish TAVI registry (2008–2020) reported in-hospital mortality of 3.4%, reflecting rates similar to those observed in the earliest years (2013–2016) of the SWEDEHEART cohort.^17^ The French TAVI registry (2009–2019) demonstrated a more pronounced trend of in-hospital mortality rates (12.7–1.9%) over time compared to our cohort (2.7–1.0%) but with a significantly higher pacemaker implantation rates up to 16.7%.^18^ Procedural risk scores, such as EuroSCORE II, decreased over time in most cohorts, aligning with the global shift with TAVI being increasingly used to treat lower risk patients.^6^^,^^16^^,^^17^^,^^19^^,^^20^ Most studies, including ours, report a median age in TAVI patients ranging from 84 to 81 years, highlighting that the procedure remains primarily utilized in older patients in European centres. The proportion of TAVI in patients <65 years accounted for 2.5% of all TAVI procedures in our study which contrasts with reports from the USA showing that TAVI is increasingly being used in younger patients, some reports showing that nearly 50% of patients <65 years now undergo TAVI.^21^

The decline in median surgical risk scores (EuroSCORE II and STS-PROM) in the TAVI cohort likely reflects a combination of factors, including the expansion of TAVI into lower-risk populations and improved granularity in contemporary risk stratification models may have enabled more precise classification of patients by operative risk.

The rising proportion of bicuspid aortic valves in both TAVI and SAVR cohorts may similarly reflect advancements in diagnostic precision. The routine use of ECG-gated CT angiography and standardized TAVI imaging protocols has improved the detection and classification of valve morphology. In parallel, heightened clinical awareness of bicuspid disease likely contributes to more systematic evaluation and documentation during preoperative assessment.

The decline in complications in TAVI-related is likely multifactorial. Advances in valve design and delivery systems have enhanced procedural precision and reduced mechanical complications. Operator experience has matured, contributing to more consistent and safer outcomes. Furthermore, there has been considerable progress in the preoperative identification of high-risk patients, allowing for better-tailored management strategies and more informed treatment selection.^22^ However, we believe that patient selection is the principal driver of the improved outcomes observed among TAVI patients. This is supported by the reduction in pre-procedural risk, as reflected by EuroSCORE II and STS-PROM, together with the absence of a consistent improvement in outcomes after adjustment for EuroSCORE II and sex, except in some later calendar years, and by the lack of improvement among high-risk patients (Supplementary Table 8–9, Supplementary Figure 5–6). Furthermore, when stratifying by risk categories, survival improved primarily in intermediate-risk patients, whereas no improvement in high-risk patients and only short-term improvement for low-risk patients (Supplementary Figure 5). This pattern might suggest that the overall improvement in survival may partly reflect the growing proportion of intermediate-risk patients. However, the results should be interpreted with caution as they are unadjusted and cannot establish causality. It remains likely that collectively these developments, improved device technology, refined procedural techniques, enhanced complication management, and better risk stratification, have all contributed to the overall decline in adverse outcomes over time in TAVI patients.

Although 5-year mortality has improved over time, it remains notably high at 38.7% for TAVI patients compared to 13% for SAVR patients. This difference likely reflects the older age and greater comorbidity of TAVI patients (median 81 years vs 71 years) rather than treatment futility. As demonstrated in previous studies, patients managed conservatively fare even worse. The observed outcomes are therefore consistent with the limited life expectancy of this population.^23^

The aim of this study was not to compare SAVR and TAVI or determine which approach is superior, but rather to highlight the shift in clinical practice over the past decade. While numerous studies have documented the increasing adoption and improved outcomes of TAVI, fewer have examined long-term trends in SAVR. It is important to remember that SAVR is associated with a very high 10-year survival of approximately 90% in younger low-risk patients, which underscores the continued relevance of SAVR in clinical practice.^24^ Despite the sharp rise in TAVI procedures, it is reassuring that annual SAVR volumes have remained stable over time, indicating high guideline adherence.

We observed a numerically higher bleeding rate after 2018. This trend can most likely be attributed to a change in the registry. In 2019, the Swedish Cardiac Surgery Registry began collecting data on the number of blood transfusion units administered during hospital stay, a variable we used as a proxy for bleeding in our analysis. This improved data granularity likely enhanced complication reporting, contributing to the apparent increase in bleeding rates.

The observed change in complication rates among SAVR patients is noteworthy. We believe this trend is partly driven by a shift in patient selection, as the widespread adoption of TAVI has offered an alternative for patients with elevated surgical risk or complex comorbidities. Consequently, the contemporary SAVR population may increasingly represent a more focused and suitable surgical cohort. In parallel with TAVI, there has also been significant advancement in preoperative evaluation, including improved imaging, risk stratification, and multidisciplinary heart team assessments, which allow for better identification of optimal candidates for surgery. This refined patient selection likely contributes to reduced perioperative complications and improved outcomes.

Concomitant coronary revascularization differed markedly between modalities (31–35% SAVR + CABG vs 7–12% TAVI + PCI). This gap likely reflects case selection, and patients with left main or multivessel CAD, diffuse/complex anatomy, or concomitant surgical indications are preferentially referred to SAVR where complete revascularization with CABG is feasible. Future analyses stratified by CAD complexity and revascularization strategy would help clarify strategy differences. However, this is outside the scope of the current study. The increase in M-SAVR may also be a result of refinement in patient selection, where younger individuals and those with longer life expectancy are more often referred for SAVR. In this population, mechanical valves offer a distinct advantage by reducing the need for future reinterventions associated with structural valve deterioration. As the lifetime management of bioprosthetic valves becomes increasingly complex in younger patients, M-SAVR represents a durable and evidence-based alternative for ensuring long-term valve function and minimizing the risk of reoperation.^25^^,^^26^ The observed decline in preprocedural risk scores, such as EuroSCORE II and STS-PROM, further suggests that the SAVR population is healthier today than a decade ago, which likely plays an important role in the favourable trajectory of complication rates and survival.

TAVI has significantly expanded access to life-saving prosthetic valve implantation, particularly for patients previously deemed inoperable, those at high surgical risk, elderly patients and frail individuals with moderate or low risk. While it is reassuring that younger, low-risk patients are still frequently treated with SAVR, the continued expansion of TAVI into younger, lower-risk populations and patients with longer life expectancy has made lifetime management one of the most pressing challenges in the field. Concerns regarding long-term TAVI valve durability and the risk of coronary obstruction during future interventions are gradually being addressed, with emerging data showing outcomes increasingly comparable to those of surgical bio prosthesis. The PARTNER 3 trial, comparing TAVI with SAVR in low-risk patients, recently reported 5-year outcomes demonstrating no significant difference between the two interventions.^27^ Similarly, the NOTION trial reported no difference in 10-year outcomes between TAVI and SAVR in low-risk patients. In contrast, observational studies have suggested higher long-term mortality among TAVI patients compared with SAVR, underscoring the need for additional long-term data.^28^ Ten-year results, expected to be published next year, will provide further insight.

Ongoing very long-term follow-up from large, randomized trials will be essential to provide definitive insights into valve performance, structural durability, and reintervention rates. These findings will be particularly important for guiding treatment decisions in patients with long life expectancy. Importantly, low surgical risk is not synonymous with young age, and age alone should neither exclude TAVI nor justify its use. Instead, decisions should be guided by anatomical suitability, comorbidities, and anticipated need for future interventions. Shared decision-making that aligns patient preferences with clinical objectives remains fundamental. As both SAVR and TAVI technologies advance, individualized lifetime planning becomes increasingly important. Redo-TAVI poses specific risks, particularly coronary obstruction, making pre-procedural planning such as CT imaging, valve choice, and commissural alignment critical. While similar risks may occur after SAVR, they are less common due to the removal of the native valve and the typically lower frame height of surgical prostheses, which reduce obstruction risk. Moreover, TAVI's minimally invasive nature limits the ability to address concomitant cardiac pathologies requiring surgical correction such as aortic aneurysms or extensive 3-vessel coronary disease.^4^

No single study can fully account for the complexity of anatomical variation, disease progression, or vascular access constraints. As the field evolves, carefully balancing the advantages of early intervention with the long-term risks of multiple procedures will be key, particularly for younger, low-risk patients who may be hesitant to undergo open-heart surgery. These clinical and personal factors must be weighed thoughtfully to ensure optimal, individualized care over the patient's lifetime.

This study provides valuable insights into real-world trends and outcomes that can help guide clinical practice. It is important to recognize certain limitations that should be considered when interpreting the results. First and foremost, this study is retrospective and observational, which inherently introduces potential biases, including selection bias related to both patient and operator decision-making. The registry cannot capture Heart-Team rationale for modality choice, nor the total denominator of patients with severe AS. Data on Ross procedures are not available in SCSR and therefore not discussed. As SWEDEHEART captures only treated prosthetic aortic valve implantation cases and not medically managed or inoperable severe AS, we cannot infer population-level treatment rates for all patients with AS. Treatment allocation was not randomized, and as such, the choice between TAVI and SAVR has been influenced by clinical judgement, institutional protocols, or perceived procedural risk, factors that may not be fully captured in the registry data. Consequently, while the study provides insights into temporal trends and outcomes, it does not permit causal conclusions. Interpretations regarding the drivers behind improvements in management strategies for aortic stenosis should therefore be viewed as exploratory and hypothesis-generating. Secondly, all complications are self-reported by the operators and are not reviewed centrally which might result in bias leading to lower complication rates. Reporting centres do however assess their complications every month to enhance credibility. Pacemaker implantation rates for SAVR were only tracked from 2017 onwards, limiting the ability to perform comprehensive temporal comparisons. For the TAVI group, four centres did not report any pacemaker implantations 2016, for which we cannot find any logical reason for. To make a fair comparison, survival analyses were restricted to patients with complete follow up in the 3- and 5-year outcome analyses. We lacked information on patients with severe aortic stenosis managed medically or deemed inoperable, potentially underestimating the broader impact of advancements in aortic stenosis management. Late complications, such as valve degeneration or endocarditis, were not evaluated, which may be critical for assessing the long-term outcomes of prosthetic aortic valve implantation, particularly for TAVI. We lacked data on cardiovascular mortality which would have added to the study as, patients undergoing TAVI who are older and often more frail, have competing risk of other comorbidities.

### Conclusion

The landscape of prosthetic aortic valve implantation has transformed over the past years, largely owing to the widespread adoption of TAVI, which has made treatment accessible to a broader range of patients with aortic stenosis. Despite the growing adoption of TAVI, SAVR utilization has remained relatively stable over the study period, with outcomes improving steadily for both procedures. As management strategies continue to evolve, sustained innovation, high-quality research, and a continuous commitment to individualized care remain essential to optimize outcomes and meet the complex, evolving challenges of this condition.

## Contributors

JT and MM conceptualised and designed the study. JT performed the data analysis, prepared tables and figures and drafted the original manuscript. MM had access to and verified the underlying data, supervised the analysis and contributed to writing, review and editing. SK, TY, OA, HB, SN, ÖF, JB, SJ, BR, PG, AL, HH, AR and MG contributed to data interpretation, critical revision of the manuscript and approved the final version for submission.

## Data sharing statement

The data underlying this article cannot be shared publicly due to privacy regulations and legal restrictions associated with the SWEDEHEART registry. As a protected national registry, individual-level data are considered sensitive personal information and cannot be made publicly available. Data access is strictly regulated and requires ethical approval in accordance with Swedish data protection laws. Therefore, the dataset used in this study is not available for public sharing.

## Declaration of interests

All authors have completed the ICMJE disclosure form. AR reports consulting for Boston Scientific, Edwards Lifesciences, Anteris and research support from Boston Scientific. All other authors declare no competing interests or financial relationships with any organisations that might have an interest in the submitted work. No authors have other relationships or activities that could appear to have influenced the submitted work.
