# Supplementary material for: Percutaneous and surgical management of aortic stenosis in the SWEDEHEART registry (2013–2023): a nationwide observational study
Source: Lancet Reg Health Eur. 2025 Nov 3;60:101520. doi: 10.1016/j.lanepe.2025.101520 (PMC12630323; doi:10.1016/j.lanepe.2025.101520)
Supplement: Supplementary Tables and Figures [file mmc1.docx]

Supplement

**Supplementary table 1. Inclusion and exclusion criteria.**

| Inclusion TAVI | Exclusion TAVI | Inclusion SAVR | Exclusion SAVR |
| --- | --- | --- | --- |
| Main indication for TAVI | TAVI done before 2013 and after 2023 | Main indication for surgery aortic stenosis | SAVR as rescue after failed TAVI |
| First TAVI only | Not having a Swedish social security number. | Concomitant CABG and/or Atri Clip | Major concomitant surgery such as other valve surgery, aortic root surgery. |
| Concomitant PCI 90days before or after. | Not defined if a new valve had been implanted. |  | Active endocarditis |
|  |  |  | Main indication for surgery other than aortic stenosis. |
|  |  |  | SAVR done before 2013 and after 2023 |

**Supplementary table 2. Outcome definitions.**

| Outcome | Definition TAVI | Definition SAVR |
| --- | --- | --- |
| Bleeding | Defined according to the SWENTRY registry as either intervention-related bleeding requiring transfusion or other intervention, or bleeding occurring during hospitalization that required intervention. Vascular complication requiring surgery other than directly correlated to the valve. Major bleeding requiring hemodynamic support or more than two units of blood. Heart tamponade during procedure or in the ward. | Defined according to the HKIR registry as bleeding requiring reoperation or transfusion of 5 or more units of blood. |
| Infection | Defined as an infection requiring antibiotic treatment during the hospital stay. | Defined as an infection requiring antibiotics other than pre-procedural prophylaxis or the growth of bacteria in clinical samples (excluding contamination). |
| In-hospital mortality | Defined as confirmed death occurring on or before the date of hospital discharge. | Defined as confirmed death occurring on or before the date of hospital discharge. |
| Stroke | Defined as a neurological deficit occurring during the intervention (e.g., stroke, reversible ischemic neurological deficit (RIND), or transient ischemic attack (TIA) or a stroke occurring during the hospital stay with symptoms lasting longer than 24 hours. | Defined as circulatory neurological impairment with symptoms lasting longer than 72 hours. |
| Myocardial infarction | During the intervention, defined as a new coronary occlusion or troponin/CKMB elevation exceeding 15 or 5 times the reference limit, respectively, with symptoms of MI. After 72 hours, MI was defined as elevated troponin or CKMB levels above the reference limit in conjunction with ECG changes indicative of MI, ventricular dysfunction, sudden death, or evidence of MI at autopsy. | Defined as an occlusion in a coronary graft requiring reoperation or percutaneous coronary intervention (PCI). Or having any PCI before discharge date. |
| Pacemaker implantation | Defined as the implantation of a new pacemaker following the intervention. | Defined as the implantation of a new pacemaker following the intervention. |
| 3-point MACE | Defined as composite of in-hospital mortality, stroke or MI. | Defined as composite of in-hospital mortality, stroke or MI. |
| Any complication | Defined as the occurrence of any one of the predefined complications during the hospital stay. | Defined as the occurrence of any one of the predefined complications during the hospital stay. |

**Supplementary table 3. Temporal trends in baseline characteristics of TAVI patients.**

| Year | 2013-2014 | 2015-2016 | 2017-2018 | 2019-2020 | 2021-2022 | 2023 | p-value |
| --- | --- | --- | --- | --- | --- | --- | --- |
| N=# | N=719 | N=1,212 | N=1,892 | N=2,515 | N=3,177 | N=1,851 |  |
| Concomitant PCI | 81 (11.3%) | 108 (8.9%) | 147 (7.8%) | 227 (9.0%) | 273 (8.6%) | 125 (6.8%) | 0.005 |
| Age | 83.0 (78.0-87.0) | 82.0 (77.0-86.0) | 82.0 (77.0-85.0) | 81.0 (76.0-85.0) | 81.0 (77.0-85.0) | 81.0 (77.0-84.0) | <0.001 |
| Sex (Male) | 351 (48.8%) | 623 (51.4%) | 970 (51.3%) | 1,389 (55.2%) | 1,756 (55.3%) | 1,061 (57.3%) | <0.001 |
| Valve morphology |  |  |  |  |  |  | <0.001 |
| Bicuspid | 0 ( 0.0%) | 48 ( 4.0%) | 120 ( 6.3%) | 184 ( 7.3%) | 302 ( 9.5%) | 206 (11.1%) |  |
| Tricuspid | 0 ( 0.0%) | 540 (44.6%) | 1,768 (93.4%) | 2,324 (92.4%) | 2,864 (90.1%) | 1,571 (84.9%) |  |
| missing | 719 (100.0%) | 624 (51.5%) | 4 ( 0.2%) | 7 ( 0.3%) | 11 ( 0.3%) | 74 ( 4.0%) |  |
| Access |  |  |  |  |  |  |  |
| Femoral | 630 (87.7%) | 1,082 (89.3%) | 1,727 (91.3%) | 2,382 (94.7%) | 3,075 (96.8%) | 1,814 (98.0%) | <0.001 |
| Alternative access | 88 (12.3%) | 130 (10.7%) | 165 (8.7%) | 133 (5.3%) | 102 (3.2%) | 37 (2.0%) |  |
| Hypertension | 509 (70.8%) | 917 (75.7%) | 1,444 (76.3%) | 1,953 (77.7%) | 2,468 (77.7%) | 1,399 (78.6%) | <0.001 |
| Diabetes | 172 (23.9%) | 302 (24.9%) | 486 (25.7%) | 652 (25.9%) | 825 (26.0%) | 458 (25.7%) | 0.88 |
| Atrial fibrilliation | 261 (36.3%) | 459 (37.9%) | 714 (37.7%) | 924 (36.7%) | 1,090 (34.3%) | 594 (33.4%) | 0.015 |
| Previous PCI | 94 (13.1%) | 219 (18.1%) | 365 (19.3%) | 467 (18.6%) | 564 (17.8%) | 289 (15.6%) | <0.001 |
| Chronic pulmonary disease | 133 (18.5%) | 230 (19.0%) | 364 (19.2%) | 399 (15.9%) | 465 (14.6%) | 235 (13.2%) | <0.001 |
| Peripheral vessel disease | 115 (16.0%) | 219 (18.1%) | 342 (18.1%) | 373 (14.8%) | 408 (12.8%) | 211 (11.8%) | <0.001 |
| Previous cardiac surgery | 173 (24.1%) | 286 (23.6%) | 310 (16.4%) | 360 (14.3%) | 398 (12.5%) | 196 (10.6%) | <0.001 |
| missing | 0 ( 0.0%) | 0 ( 0.0%) | 0 ( 0.0%) | 0 ( 0.0%) | 0 ( 0.0%) | 70 ( 3.8%) |  |
| Recent MI | 42 ( 5.8%) | 75 ( 6.2%) | 74 ( 3.9%) | 78 ( 3.1%) | 93 ( 2.9%) | 51 ( 2.9%) | <0.001 |
| Pulmonary hypertension |  |  |  |  |  |  | <0.001 |
| 0-30mmhg | 114 (18.1%) | 193 (19.7%) | 316 (21.5%) | 463 (23.9%) | 643 (26.3%) | 420 (32.0%) |  |
| 31-55mmhg | 396 (63.0%) | 623 (63.6%) | 948 (64.4%) | 1,243 (64.1%) | 1,541 (63.0%) | 778 (59.3%) |  |
| 56-120mmhg | 119 (18.9%) | 164 (16.7%) | 207 (14.1%) | 232 (12.0%) | 261 (10.7%) | 113 ( 8.6%) |  |
| unknown | 0 ( 0.0%) | 0 ( 0.0%) | 0 ( 0.0%) | 0 ( 0.0%) | 2 ( 0.1%) | 0 ( 0.0%) |  |
| NYHA Class |  |  |  |  |  |  | <0.001 |
| Class I | 2 ( 0.3%) | 16 ( 1.3%) | 92 ( 4.9%) | 38 ( 1.5%) | 57 ( 1.8%) | 56 ( 3.1%) |  |
| Class II | 58 ( 8.1%) | 145 (12.0%) | 273 (14.4%) | 564 (22.4%) | 823 (25.9%) | 545 (30.6%) |  |
| Class III | 526 (73.2%) | 884 (72.9%) | 1,334 (70.5%) | 1,639 (65.2%) | 2,009 (63.2%) | 1,032 (57.9%) |  |
| Class IIII | 133 (18.5%) | 162 (13.4%) | 193 (10.2%) | 271 (10.8%) | 287 ( 9.0%) | 146 ( 8.2%) |  |
| missing | 0 ( 0.0%) | 5 ( 0.4%) | 0 ( 0.0%) | 3 ( 0.1%) | 1 ( 0.0%) | 2 ( 0.1%) |  |
| EuroSCORE II | 5.6 (3.3-10.2) | 4.9 (3.0-9.0) | 4.1 (2.4-7.0) | 3.3 (2.0-5.9) | 3.1 (1.9-5.5) | 2.7 (1.7-4.6) | <0.001 |
| STS-PROM | 3.3 (1.9-4.1) | 2.5 (1.5-4.3) | 2.1 (1.3-3.8) | 1.9 (1.2-3.2) | 1.6 (1.1-2.9) | 1.6 (1.1-2.8) | <0.001 |
| Left ventricular ejection fraction |  |  |  |  |  |  | <0.001 |
| LVEF>50% | 434 (60.4%) | 733 (60.5%) | 1,263 (66.8%) | 1,842 (73.6%) | 2,366 (75.0%) | 1,347 (76.3%) |  |
| LVEF 31%–50% | 118 (16.4%) | 206 (17.0%) | 323 (17.1%) | 430 (17.2%) | 547 (17.3%) | 292 (16.5%) |  |
| LVEF 21%–30% | 98 (13.6%) | 176 (14.5%) | 204 (10.8%) | 170 ( 6.8%) | 184 ( 5.8%) | 102 ( 5.8%) |  |
| LVEF 20% or less | 69 ( 9.6%) | 97 ( 8.0%) | 101 ( 5.3%) | 61 ( 2.4%) | 58 ( 1.8%) | 24 ( 1.4%) |  |
| TAVI+PCI with LVEF <20% | 11 (1.5%) | 16 (1.3%) | 15 (0.8% | 13 (0.5%) | 9 (0.2%) | 5 (0.2%) | <0.001 |
| New permanent pacemaker | 97 (13.5%) | 117 ( 9.7%) | 132 ( 7.0%) | 218 ( 8.7%) | 215 ( 6.8%) | 136 ( 7.3%) | <0.001 |
| Bleeding | 99 (13.8%) | 127 (10.5%) | 147 ( 7.8%) | 159 ( 6.3%) | 141 ( 4.4%) | 93 ( 5.0%) | <0.001 |
| Stroke | 22 ( 3.1%) | 23 ( 1.9%) | 31 ( 1.6%) | 49 ( 1.9%) | 51 ( 1.6%) | 35 ( 1.9%) | 0.19 |
| MI | 8 ( 1.1%) | 16 ( 1.3%) | 12 ( 0.6%) | 23 ( 0.9%) | 32 ( 1.0%) | 12 ( 0.6%) | 0.31 |
| In-hospital all-cause death | 26 ( 3.6%) | 22 ( 1.8%) | 30 ( 1.6%) | 22 ( 0.9%) | 31 ( 1.0%) | 18 ( 1.0%) | <0.001 |
| Any complication | 210 (29.2%) | 218 (18.0%) | 291 (15.4%) | 397 (15.8%) | 403 (12.7%) | 244 (13.2%) | <0.001 |

**Supplementary table 4. Temporal trends in baseline characteristics of SAVR patients.**

| Year | 2013-2014 | 2015-2016 | 2017-2018 | 2019-2020 | 2021-2022 | 2023 | p-value |
| --- | --- | --- | --- | --- | --- | --- | --- |
| N=# | N=1,921 | N=2,091 | N=2,045 | N=1,603 | N=1,607 | N=750 |  |
| Concomittant CABG | 670 (34.9%) | 645 (30.8%) | 690 (33.7%) | 532 (33.2%) | 522 (32.5%) | 233 (31.1%) | 0.095 |
| Age | 73.0 (66.0-78.0) | 72.0 (66.0-78.0) | 72.0 (66.0-76.0) | 71.0 (65.0-75.0) | 70.0 (64.0-74.0) | 70.0 (64.0-74.0) | <0.001 |
| Sex (Male) | 1,189 (61.9%) | 1,329 (63.6%) | 1,351 (66.1%) | 1,135 (70.8%) | 1,137 (70.8%) | 529 (70.5%) | <0.001 |
| Valve Morphology |  |  |  |  |  |  | <0.001 |
| Bicuspid | 0 ( 0.0%) | 0 ( 0.0%) | 0 ( 0.0%) | 536 (33.4%) | 529 (32.9%) | 277 (36.9%) | 0.021 |
| Tricuspid | 0 ( 0.0%) | 0 ( 0.0%) | 5 ( 0.2%) | 1,035 (64.6%) | 1,046 (65.1%) | 452 (60.3%) |  |
| Other | 0 ( 0.0%) | 0 ( 0.0%) | 0 ( 0.0%) | 18 ( 1.1%) | 20 ( 1.2%) | 14 ( 1.9%) |  |
| missing | 1,921 (100.0%) | 2,091 (100.0%) | 2,040 (99.8%) | 14 ( 0.9%) | 12 ( 0.7%) | 7 ( 0.9%) |  |
| Hypertension | 1 ( 0.1%) | 590 (28.2%) | 1,232 (60.2%) | 1,145 (71.4%) | 1,183 (73.6%) | 565 (75.5%) | <0.001 |
| missing | 1,920 (99.9%) | 1,241 (59.3%) | 307 (15.0%) | 5 ( 0.3%) | 2 ( 0.1%) | 0 ( 0.0%) |  |
| Diabetes | 376 (19.7%) | 462 (22.1%) | 447 (21.9%) | 418 (26.1%) | 436 (27.1%) | 195 (26.1%) | <0.001 |
| Atrial fibrilliation | 209 (18.1%) | 340 (17.0%) | 366 (18.3%) | 291 (18.6%) | 269 (17.1%) | 129 (17.4%) | 0.76 |
| Previous PCI | 81 ( 4.2%) | 116 ( 5.5%) | 147 ( 7.2%) | 110 ( 6.9%) | 111 ( 6.9%) | 51 ( 6.8%) | <0.001 |
| Chronic pulmonary disease | 185 ( 9.7%) | 184 ( 8.8%) | 152 ( 7.4%) | 119 ( 7.4%) | 135 ( 8.4%) | 63 ( 8.4%) | <0.001 |
| Peripheral vessel disease | 127 ( 6.7%) | 115 ( 5.5%) | 105 ( 5.1%) | 66 ( 4.1%) | 65 ( 4.0%) | 28 ( 3.7%) | <0.001 |
| Previous cardiac surgery | 56 ( 2.9%) | 39 ( 1.9%) | 52 ( 2.5%) | 37 ( 2.3%) | 41 ( 2.6%) | 15 ( 2.0%) | <0.001 |
| missing | 15 ( 0.8%) | 1 ( 0.0%) | 2 ( 0.1%) | 1 ( 0.1%) | 0 ( 0.0%) | 0 ( 0.0%) |  |
| Recent MI | 160 ( 8.4%) | 168 ( 8.0%) | 141 ( 6.9%) | 83 ( 5.2%) | 105 ( 6.5%) | 40 ( 5.3%) | <0.001 |
| Pulmonary hypertension |  |  |  |  |  |  | <0.001 |
| 0-30mmhg | 1,462 (82.6%) | 1,710 (81.8%) | 1,709 (83.7%) | 1,249 (77.9%) | 1,242 (77.3%) | 585 (78.0%) |  |
| 31-55mmhg | 260 (14.7%) | 327 (15.6%) | 290 (14.2%) | 190 (11.9%) | 197 (12.3%) | 113 (15.1%) |  |
| 56-120mmhg | 48 ( 2.7%) | 53 ( 2.5%) | 44 ( 2.2%) | 26 ( 1.6%) | 34 ( 2.1%) | 10 ( 1.3%) |  |
| unknown | 0 ( 0.0%) | 0 ( 0.0%) | 0 ( 0.0%) | 138 ( 8.6%) | 134 ( 8.3%) | 42 ( 5.6%) |  |
| NYHA Class |  |  |  |  |  |  | <0.001 |
| Class I | 165 ( 8.7%) | 189 ( 9.0%) | 126 ( 6.2%) | 110 ( 6.9%) | 132 ( 8.2%) | 71 ( 9.5%) |  |
| Class II | 738 (38.8%) | 820 (39.2%) | 906 (44.4%) | 741 (46.2%) | 737 (45.9%) | 380 (50.7%) |  |
| Class III | 903 (47.5%) | 1,005 (48.1%) | 951 (46.6%) | 696 (43.4%) | 644 (40.1%) | 276 (36.8%) |  |
| Class IIII | 63 ( 3.3%) | 66 ( 3.2%) | 53 ( 2.6%) | 42 ( 2.6%) | 73 ( 4.5%) | 18 ( 2.4%) |  |
| missing | 31 ( 1.6%) | 10 ( 0.5%) | 6 ( 0.3%) | 14 ( 0.9%) | 21 ( 1.3%) | 5 ( 0.7%) |  |
| EuroSCORE II | 1.5 (1.0-2.3) | 1.4 (1.0-2.2) | 1.4 (1.0-2.2) | 1.4 (1.0-2.2) | 1.4 (1.0-2.2) | 1.3 (0.9-2.1) | <0.001 |
| STS-PROM | 1.8 (1.2-3.0) | 1.7 (1.1-2.7) | 1.6 (1.1-2.6) | 1.7 (1.0-2.7) | 1.6 (1.0-2.5) | 1.6 (1.1-2.6) | <0.001 |
| Left ventricular ejection fraction |  |  |  |  |  |  | <0.001 |
| LVEF>50% | 1,459 (76.4%) | 1,620 (77.5%) | 1,619 (79.2%) | 1,325 (82.7%) | 1,238 (77.0%) | 598 (79.7%) |  |
| LVEF 31%–50% | 352 (18.4%) | 394 (18.9%) | 345 (16.9%) | 223 (13.9%) | 284 (17.7%) | 109 (14.5%) |  |
| LVEF 21%–30% | 77 ( 4.0%) | 63 ( 3.0%) | 59 ( 2.9%) | 34 ( 2.1%) | 41 ( 2.6%) | 28 ( 3.7%) |  |
| LVEF 20% or less | 21 ( 1.1%) | 13 ( 0.6%) | 20 ( 1.0%) | 18 ( 1.1%) | 32 ( 2.0%) | 15 ( 2.0%) |  |
| missing | 0 ( 0.0%) | 0 ( 0.0%) | 0 ( 0.0%) | 3 ( 0.2%) | 12 ( 0.7%) | 0 ( 0.0%) |  |
| SAVR+CABG with LVEF <20% | 8 (0.4%) | 9 (0.4%) | 8 (0.4%) | 1 (<0.01%) | 5 (0.3%) | 9 (1.2%) |  |
| New permanent pacemaker | 0 (0%) | 8 ( 2.6%) | 74 ( 4.2%) | 60 ( 3.7%) | 77 ( 4.8%) | 31 ( 4.1%) | 0.39 |
| Bleeding | 123 ( 6.4%) | 101 ( 4.8%) | 110 ( 5.4%) | 112 ( 7.0%) | 126 ( 7.8%) | 56 ( 7.5%) | 0.001 |
| Stroke | 46 ( 2.4%) | 36 ( 1.7%) | 33 ( 1.6%) | 24 ( 1.5%) | 29 ( 1.8%) | 14 ( 1.9%) | 0.40 |
| MI | 10 ( 0.5%) | 16 ( 0.8%) | 17 ( 0.8%) | 10 ( 0.6%) | 13 ( 0.8%) | 8 ( 1.1%) | 0.70 |
| In-hospital all-cause death | 30 ( 1.6%) | 23 ( 1.1%) | 22 ( 1.1%) | 5 ( 0.3%) | 9 ( 0.6%) | 3 ( 0.4%) | <0.001 |
| Any complication | 354 (18.4%) | 290 (13.9%) | 352 (17.2%) | 256 (16.0%) | 305 (19.0%) | 140 (18.7%) | <0.001 |

**Supplementary table 5. Valve-in-Valve procedures per year and type of Valve-in-Valve.**

| Year | 2013 | 2014 | 2015 | 2016 | 2017 | 2018 | 2019 | 2020 | 2021 | 2022 | 2023 |
| --- | --- | --- | --- | --- | --- | --- | --- | --- | --- | --- | --- |
| N=# | 19 | 18 | 29 | 15 | 21 | 19 | 16 | 17 | 21 | 17 | 32 |
| Valve-in-valve |  |  |  |  |  |  |  |  |  |  |  |
| SAVR-SAVR | 8 (0.7%) | 8 (0.5%) | 15 (0.9%) | 3 (0.2%) | 5 (0.3%) | 1 (<1%) | 0 (0.0%) | 1 (<1%) | 1 (<1%) | 0 (0.0%) | 0 (0.0%) |
| SAVR-TAVI | 0 (0.0%) | 1 (0.1%) | 1 (0.1%) | 0 (0.0%) | 0 (0.0%) | 5 (0.2%) | 3 (0.1%) | 8 (0.4%) | 14 (0.6%) | 8 (0.3%) | 25 (1.0%) |
| TAVI-SAVR | 0 (0.0%) | 2 (0.1%) | 2 (0.1%) | 2 (0.1%) | 1 (0.1%) | 0 (0.0%) | 1 (<1%) | 0 (0.0%) | 1 (<1%) | 1 (<1%) | 1 (<1%) |
| TAVI-TAVI | 11 (0.9%) | 7 (0.5%) | 11 (0.7%) | 10 (0.6%) | 15 (0.8%) | 13 (0.6%) | 12 (0.6%) | 8 (0.4%) | 5 (0.2%) | 6 (0.2%) | 6 (0.2%) |

**Supplementary table 6. Comparison of different TAVI and SAVR cohorts.**

| Study  (Publication year) | Included years  Number of patients | Patient characteristics | In-hospital outcomes | 30-day all-cause mortality  1-year all-cause mortality  3-year all-cause mortality  5-year all-cause mortality |
| --- | --- | --- | --- | --- |
| SWEDEHEART  TAVI cohort  (2025) | 2013-2023  11,678 patients | Age:83 - 81  Diabetes: 23.9% - 25.7%  CPD: 18.5% - 13.2%  STS-PROM: 2.7 – 1.6  EuroSCORE II: 5.6 – 2.7 | In-hospital mortality:10.5% - 1.3%  PM: 12.1% - 7.4%  Stroke: 2.9% 1.9% | 2023: 4.6% - 1.6%  2023: 10.8% - 6.9%  2021: 29.7% - 22.8%  2019: 55.3% - 38.7% |
| SWEDEHEART  SAVR cohort  (2025) | 2013-2023  11,769 patients | Age: 73 - 70  Diabetes: 19.7% - 26.1%  CPD: 9.7% - 8.4%  STS-PROM: 1.9 – 1.7  EuroSCORE II: 1.5 – 1.3 | In-hospital mortality: 1.7% - 0.5%  PM: 4.1% - 5.0%  Stroke: 2.9% - 2.3% | 2023: 2.1% - 0.9%  2023: 5.0% - 2.2%  2021: 10.5% - 7.0%  2019: 18.1% - 12.6% |
| STS-ACC-TVT: (30)  (2020) | 2011-2019  276,316 | Age: 84 - 80  Diabetes: N/A  COPD: N/A  STS-PROM: 6.91 – 4.38  EuroSCORE II: N/A | In-hospital mortality: 5.36% - 1.31%  PM: 9.10% - 8.34%  Stroke: 2.10% - 1.59% | 24.29% - 12.55%  N/A  N/A |
| DANISH TAVI:(22)  (2022) | 2008-2020  6,097 | Age: 82 - 81  Diabetes: 14.2% - 18.8%  COPD: 16.0% - 14.0%  STS-PROM: N/A  EuroSCORE II: N/A | N/A | 90-day mortality: 3.4%  N/A  N/A |
| FRANCE TAVI: (11)  (2017) | 2010-2015  16,969 | Age: 82 – 83  Diabetes: 27.3% - 26.1%  COPD: 26.1% - 15.0%  STS-PROM: N/A  Log EuroSCORE: 20.3 – 13.6 | In-hospital mortality: 8.6% - 2.7%  PM: 13.6% - 18.4%  Stroke: 1.7% - 1.6% | N/A  N/A  N/A |
| UK-TAVI(13)  (2015) | 2007-2012  3,980 | Age: 82 - 81  Diabetes: 22.9% - 22.4%  COPD: 25.7% - 24.0%  STS-PROM: N/A  EuroSCORE II: N/A | In-hospital mortality: N/A  PM: 20.3%  Stroke: 3.6% - 2.4% | 27.2% - 15.5%  46.3% - 25.8%  64.2% - 46.9% |
| GERMANY TAVI(12)  2021 | 2013-2020  15,344 | Age: 81 - 81  Diabetes: 38.1% - 34.2%  COPD: 31.1% - 18.8%  STS-PROM: 7.2 - 4.6  EuroSCORE II: 7.4 - 5.2 | In-hospital mortality: 5.5% - 1.6%  PM: 20.1% - 13.8%  Stroke: 4.0% - 3.0% | N/A  N/A  N/A |
| PMSI FRANCE TAVI cohort  2022 | 2009 – 2019  65.651 | Age: 82 - 82  Diabetes: N/A  COPD: N/A  STS-PROM: N/A  Charlson score: 1.43 – 0.73 | In-hospital mortality: 12.7% - 1.9%  PM: 11.8% - 16.7%  Stroke: 3.6% - 2.1% | N/A  N/A  N/A |
| PMSI FRANCE SAVR (23)cohort  2022 | 2007 -2019  152,767 | Age: 72 - 69  Diabetes: N/A  COPD: N/A  STS-PROM: N/A  Charlson score: 1.13 – 0.65 | In-hospital mortality: 5.0% - 1.9%  PM: 4.0% - 5.2%  Stroke: 1.5% - 1.8% | N/A  N/A  N/A |
| US SAVR*  James E Harvey | 2012 – 2019  64,973 | Age: 74  Diabetes: N/A  COPD: N/A  STS-PROM: N/A  Elixhauser comorbidity index: 4.4 | In-hospital mortality: 2.63% - 1.36%  PM: 4.94% - 5.83%  Stroke: 2.62% - 1.59% | N/A  N/A  N/A |
| US TAVI (25)*  James E Harvey | 2012 – 2019  146,239 | Age: 81  Diabetes: N/A  COPD: N/A  STS-PROM: N/A  Elixhauser comorbidity index: 6.4 | In-hospital mortality: 4.42% - 0.84%  PM: 8.95% - 9.32%  Stroke: 3.47% - 1.48% | N/A  N/A  N/A |
| US SAVR **  Sandra b Lauck | 2012 – 2019  211,246 | Age: 74.3  Diabetes: 9.1%  COPD: 26.0%  STS-PROM: N/A  EuroSCORE II: N/A  Elichauser comorbidity index: 4.4 | 30-Day Mortality: 4.8% - 4.6%  PM: N/A  Stroke: N/A | N/A  N/A  N/A |
| US TAVI(21) **  Sandra b. lauck | 2012 – 2019  179,897 | Age: 80.7  Diabetes: 19.6%  COPD: 35.1%  STS-PROM: N/A  EuroSCORE II: N/A  Elichauser comorbidity index: 6.4 | 30-Day Mortality: 6.3% - 2.0%  PM: N/A  Stroke: N/A | N/A  N/A  N/A |
| US TAVI STS/ACC TVT  Suzanne V Arnold (24) | 2019 – 2022  210,495 | Age: 80 - 79  Diabetes: 38.5% - 38.0%  COPD: N/A  STS-PROM: 3.7 – 3.0  EuroSCORE II: N/A | In-hospital Mortality: 1.5% - 1.3%  30-Day Mortality: 2.4% - 2.2%  PM: 11.8% - 9.9%  Stroke: 2.2% - 2.1% | N/A  N/A  N/A |

*Only patients without concomitant surgery were included. No comparison in temporal trends was included. **No comparison in temporal trends was included.

**Supplementary table 7. Variables utilized for calculating EuroSCORE II and STS-PROM.**

| **EuroSCORE II** | **STS-PROM** |
| --- | --- |
| Gender | Gender |
| Age | Age |
| Bmi | Weight |
| NYHA class | Height |
| Diabetes on insulin | Surgery priority |
| Peripheral vessel disease | Creatinine levels |
| Mobility | Dialysis |
| Kidney function | Hypertension |
| Critical preoperative status | Previous stroke |
| Previous MI | Peripheral vessel disease |
| Previous cardiac surgery | Previous stroke |
| Weight of surgery | Active cancer |
| Priority of surgery | Diabetes |
| Left ventricular ejection fraction | Active endocarditis |
|  | Previous PCI |
|  | NYHA class |
|  | Atrial fibrilliation |
|  | Number of diseased coronary arteries |
|  | Left main stenosis |
|  | Left ventricular ejection fraction |
|  | Preoperative inotropes |

**Supplementary table 8. Adjusted hazard ratios for 1-year all-cause mortality by calendar year for SAVR. Reference year is 2013**

| Year | Hazard ratio | 95% CI (lower-upper) | P-value |
| --- | --- | --- | --- |
| 2014 | 1.72 | 1.13-2.60 | 0.01 |
| 2015 | 0.91 | 0.59-1.41 | 0.68 |
| 2016 | 1.29 | 0.86-1.94 | 0.21 |
| 2017 | 1.02 | 0.68-1.52 | 0.94 |
| 2018 | 0.78 | 0.52-1.18 | 0.24 |
| 2019 | 0.94 | 0.64-1.39 | 0.77 |
| 2020 | 0.88 | 0.59-1.30 | 0.52 |
| 2021 | 0.98 | 0.67-1.44 | 0.92 |
| 2022 | 0.89 | 0.61-1.31 | 0.56 |
| 2023 | 0.84 | 0.57-1.24 | 0.39 |

Models adjusted for EuroSCORE II and sex. Adjusted hazard ratios for 1-year all-cause mortality in SAVR patients by calendar year, with 2013 as reference. Mortality was significantly higher in 2014 (HR 1.72, 95CI 1.13-2.60, p=0.01), while hazard rations for subsequent years did not differ significantly from 2013.

**Supplementary table 9. Adjusted hazard ratios for 1-year all-cause mortality by calendar year for TAVI. Reference year is 2013.**

| Year | Hazard ratio | 95% CI (lower-upper) | P-value |  |
| --- | --- | --- | --- | --- |
| 2014 | 1.27 | 0.84-1.93 | 0.26 |  |
| 2015 | 0.95 | 0.61-1.47 | 0.81 |  |
| 2016 | 0.63 | 0.39-1.04 | 0.07 |  |
| 2017 | 0.67 | 0.41-1.08 | 0.10 |  |
| 2018 | 0.76 | 0.48-1.20 | 0.24 |  |
| 2019 | 0.49 | 0.28-0.86 | 0.01 |  |
| 2020 | 0.43 | 0.24-0.78 | <0.01 |  |
| 2021 | 0.63 | 0.38-1.05 | 0.08 |  |
| 2022 | 0.64 | 0.38-1.07 | 0.09 |  |
| 2023 | 0.44 | 0.24-0.81 | 0.01 |  |

Models adjusted for EuroSCORE II and sex. Adjusted hazard ratios for 1-year all-cause mortality in TAVI patients by calendar year, with 2013 as reference. Risk of mortality declined significantly in 2019 (HR 0.49, 95% CI 0.28-0.86, p=0.01), 2020 (HR 0.43, 95% CI 0.24-0.78, p<0.01) and 2023 (HR 0.44, 95% CI 0.24-0.81, p=0.01) while other years showed non-significant reductions compared to 2013.

**Supplementary table 10. Adjusted hazard ratios for 30-day all-cause mortality by calendar year for TAVI. Reference year is 2013.**

| Year | Hazard ratio | 95% CI (lower-upper) | P-value |  |
| --- | --- | --- | --- | --- |
| 2014 | 1.61 | 0.85-3.04 | 0.14 |  |
| 2015 | 0.52 | 0.24-1.11 | 0.09 |  |
| 2016 | 1.06 | 0.56-2.01 | 0.86 |  |
| 2017 | 0.69 | 0.36-1.34 | 0.28 |  |
| 2018 | 0.56 | 0.28-1.08 | 0.08 |  |
| 2019 | 0.38 | 0.19-0.78 | 0.01 |  |
| 2020 | 0.42 | 0.21-0.84 | 0.01 |  |
| 2021 | 0.39 | 0.19-0.77 | 0.01 |  |
| 2022 | 0.50 | 0.27-0.94 | 0.03 |  |
| 2023 | 0.46 | 0.24-0.87 | 0.02 |  |

Models adjusted for EuroSCORE II and sex. Adjusted hazard ratios for 30-day all-cause mortality in TAVI patients by calendar year, with 2013 as reference. Risk of mortality declined significantly from 2018 and onward while earlier years showed non-significant reductions compared to 2013.

**Supplementary table 11. Adjusted hazard ratios for 30-day all-cause mortality by calendar year for SAVR. Reference year is 2013.**

| Year | Hazard ratio | 95% CI (lower-upper) | P-value |
| --- | --- | --- | --- |
| 2014 | 1.15 | 0.59-2.23 | 0.67 |
| 2015 | 0.99 | 0.50-1.94 | 0.97 |
| 2016 | 0.72 | 0.34-1.52 | 0.40 |
| 2017 | 0.89 | 0.45-1.81 | 0.76 |
| 2018 | 0.99 | 0.50-1.97 | 0.99 |
| 2019 | 0.43 | 0.17-1.07 | 0.07 |
| 2020 | 0.40 | 0.15-1.04 | 0.06 |
| 2021 | 0.76 | 0.35-1.61 | 0.47 |
| 2022 | 0.39 | 0.15-1.01 | 0.05 |
| 2023 | 0.47 | 0.19-1.18 | 0.11 |

Models adjusted for EuroSCORE II and sex. Adjusted hazard ratios for 30-day all-cause mortality in SAVR patients by calendar year, with 2013 as reference. Risk of mortality showed no significant decline for any calendar year when adjusting for EuroSCORE II and sex.

**
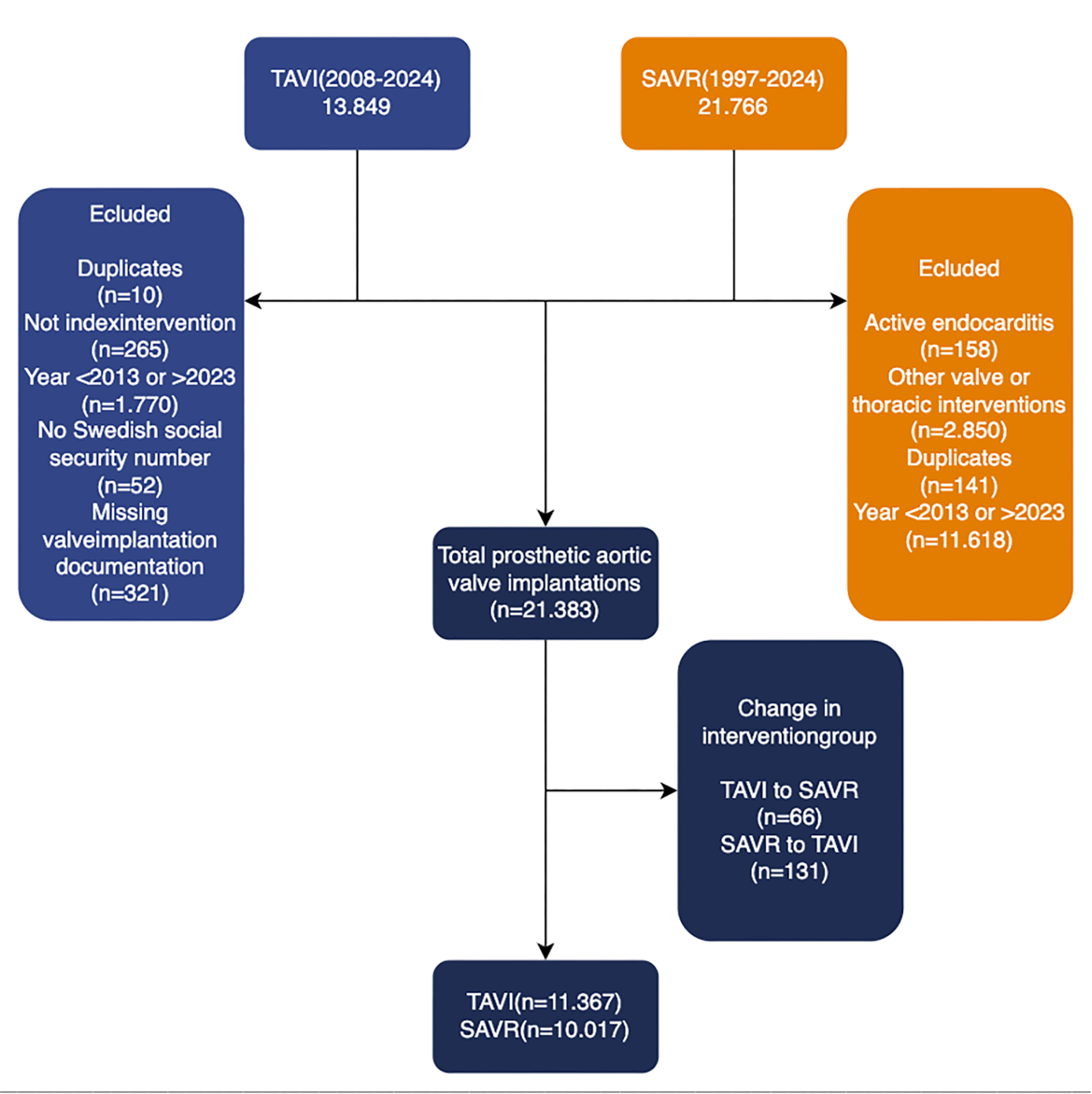
**

**Supplementary figure 1. Inclusion and exclusion criteria flowchart.**

**
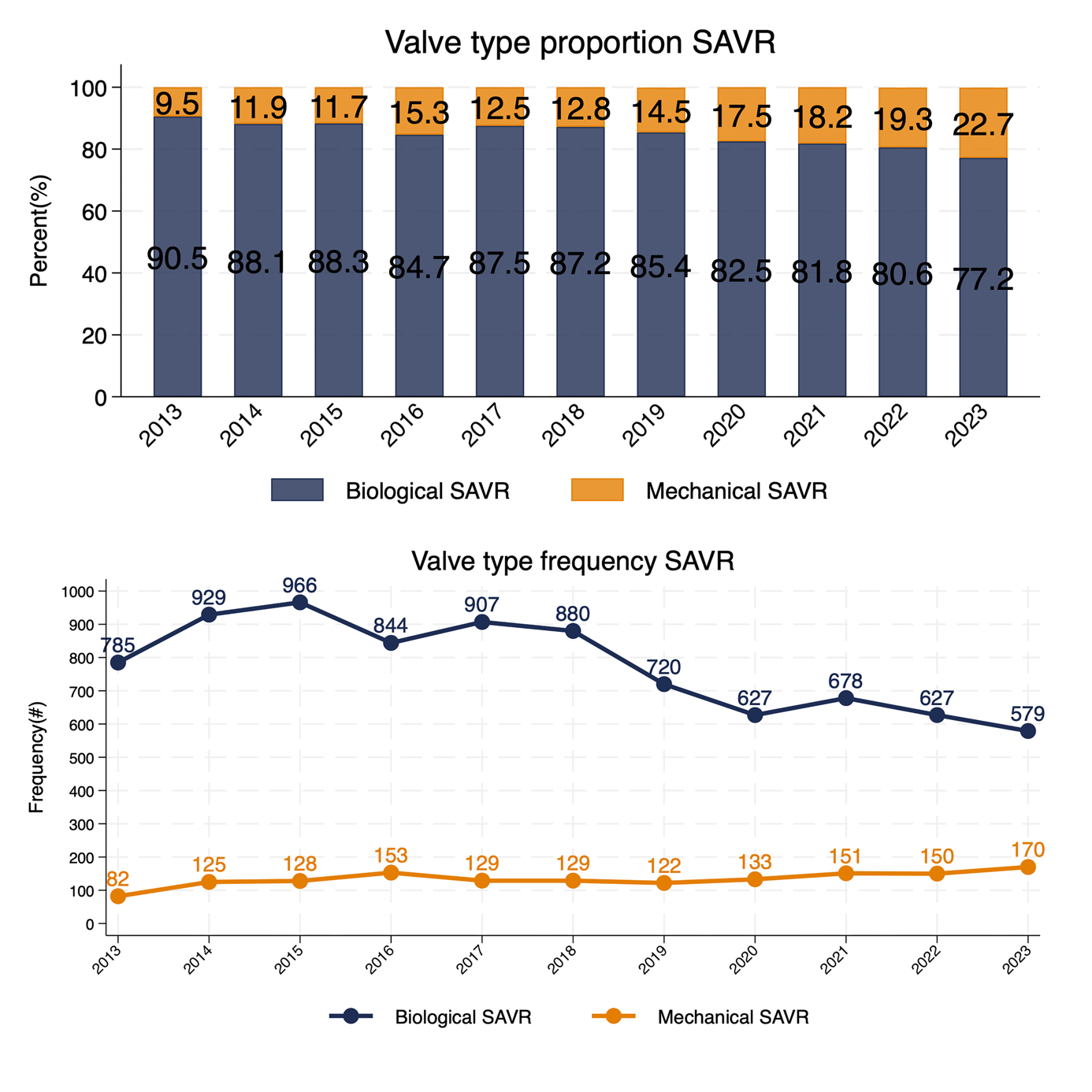
**

**Supplementary figure 2. Proportion and frequency of mechanical and biological surgical valve prosthesis over 10 years.**

(a)Distrubition of biological and mechanical SAVR from 2013 to 2023. (b) Frequency of biological and mechanical SAVR from 2013 to 2023.


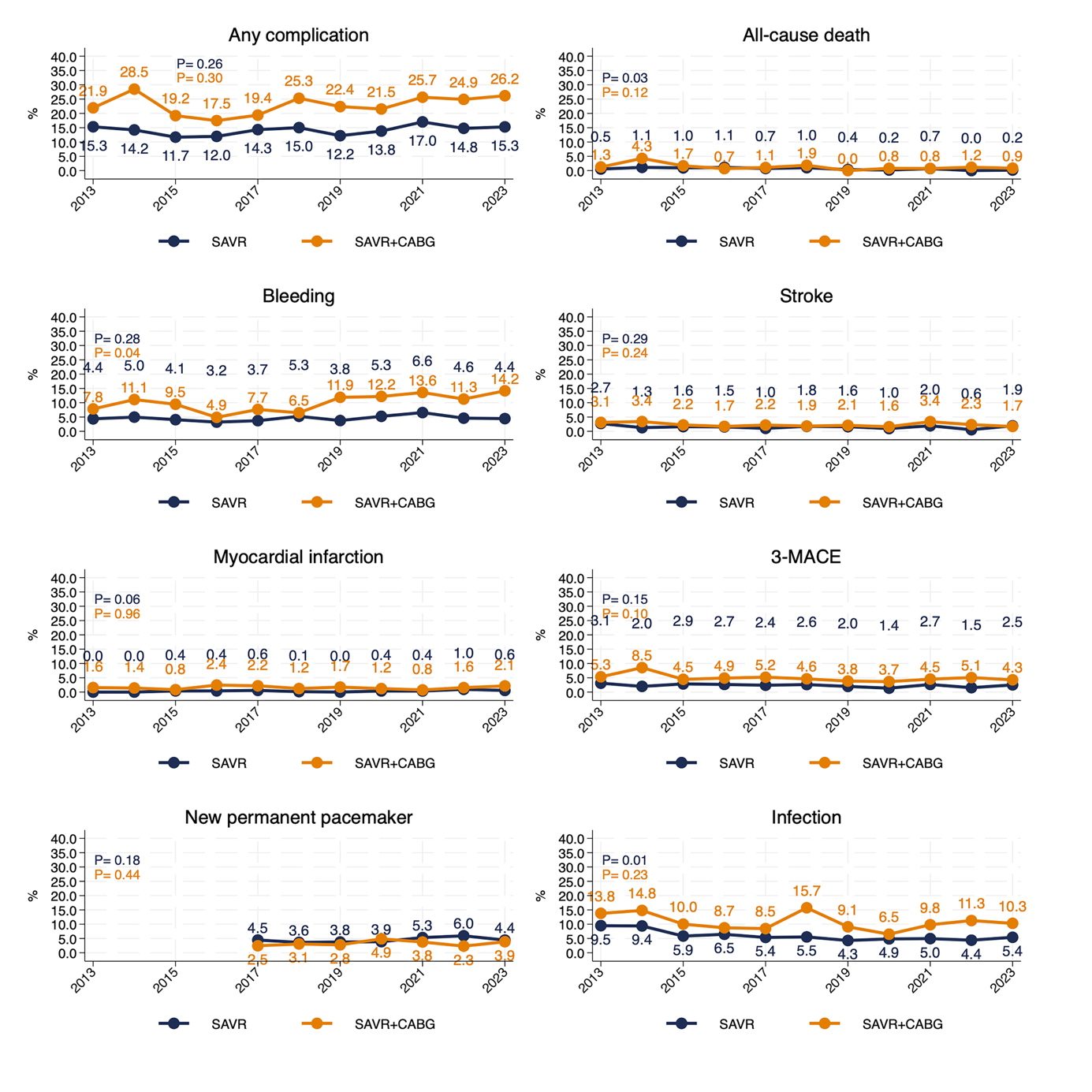


**Supplementary figure 3. Temporal Trends in In-Hospital Complications for SAVR and SAVR+CABG Patients (2013–2023)**

(a) Proportion of patients experiencing any in-hospital complication over time. (b) Proportion of patients experiencing major adverse cardiac events (MACE) during hospitalization. (c) In-hospital all-cause mortality rates over time. (d) Rates of bleeding complications during hospitalization. (e) Incidence of stroke during hospitalization. (f) Incidence of myocardial infarction during hospitalization. (g) Rates of pacemaker implantation following the procedure. (h) Incidence of infections during hospitalization. Results show higher complication risk in patients undergoing concomitant coronary surgery driven by higher risk of bleeding and infections.


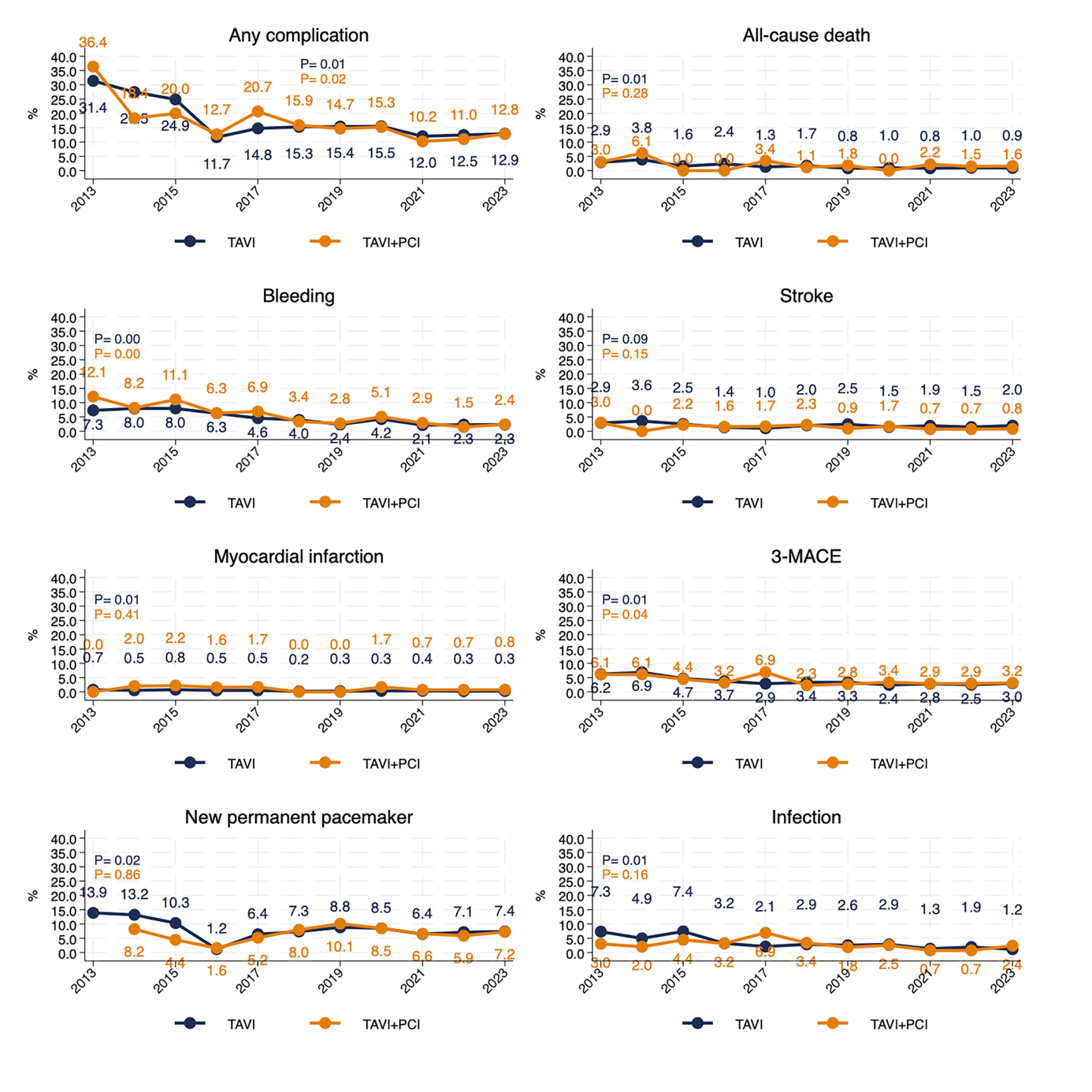


**Supplementary figure 4. Temporal Trends in In-Hospital Complications for TAVI and TAVI+PCI Patients (2013–2023)**

(a) Proportion of patients experiencing any in-hospital complication over time. (b) Proportion of patients experiencing major adverse cardiac events (MACE) during hospitalization. (c) In-hospital all-cause mortality rates over time. (d) Rates of bleeding complications during hospitalization. (e) Incidence of stroke during hospitalization. (f) Incidence of myocardial infarction during hospitalization. (g) Rates of pacemaker implantation following the procedure. (h) Incidence of infections during hospitalization. Results show similar outcomes in complications between TAVI+PCI and isolated TAVI.

**
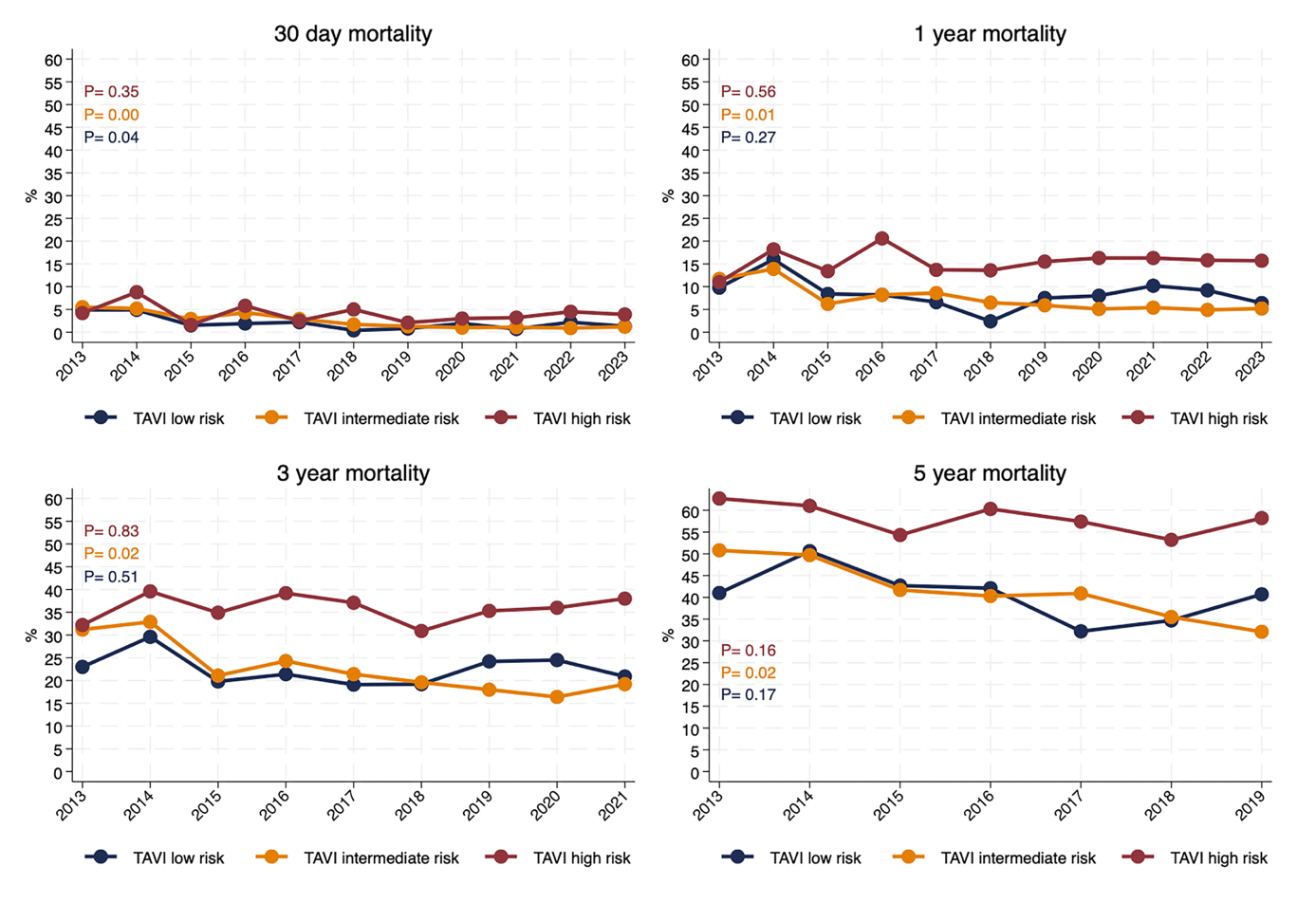
**

**Supplementary Figure 5. Temporal Trends in All-Cause Mortality at 30days, One, Three, and Five Years for Different Risk Stratifcations of TAVI Patients (2013–2023**)

(a) 30-day all-cause mortality rates over time for TAVI with low-, intermediate and high periprocedural risk (b) One-year all-cause mortality rates over time for TAVI with low-, intermediate and high periprocedural risk (c) Three-year all-cause mortality rates over time TAVI with low-, intermediate and high periprocedural risk (d) Five-year all-cause mortality rates over time for TAVI with low-, intermediate and high periprocedural risk. Significant improved mortality is seen in intermediate risk patients for all follow-up times. No significant improvement was seen for high-risk TAVI patients. Significant improvement was seen for 30-day mortality in low-risk patients, but not for longer term follow-up.


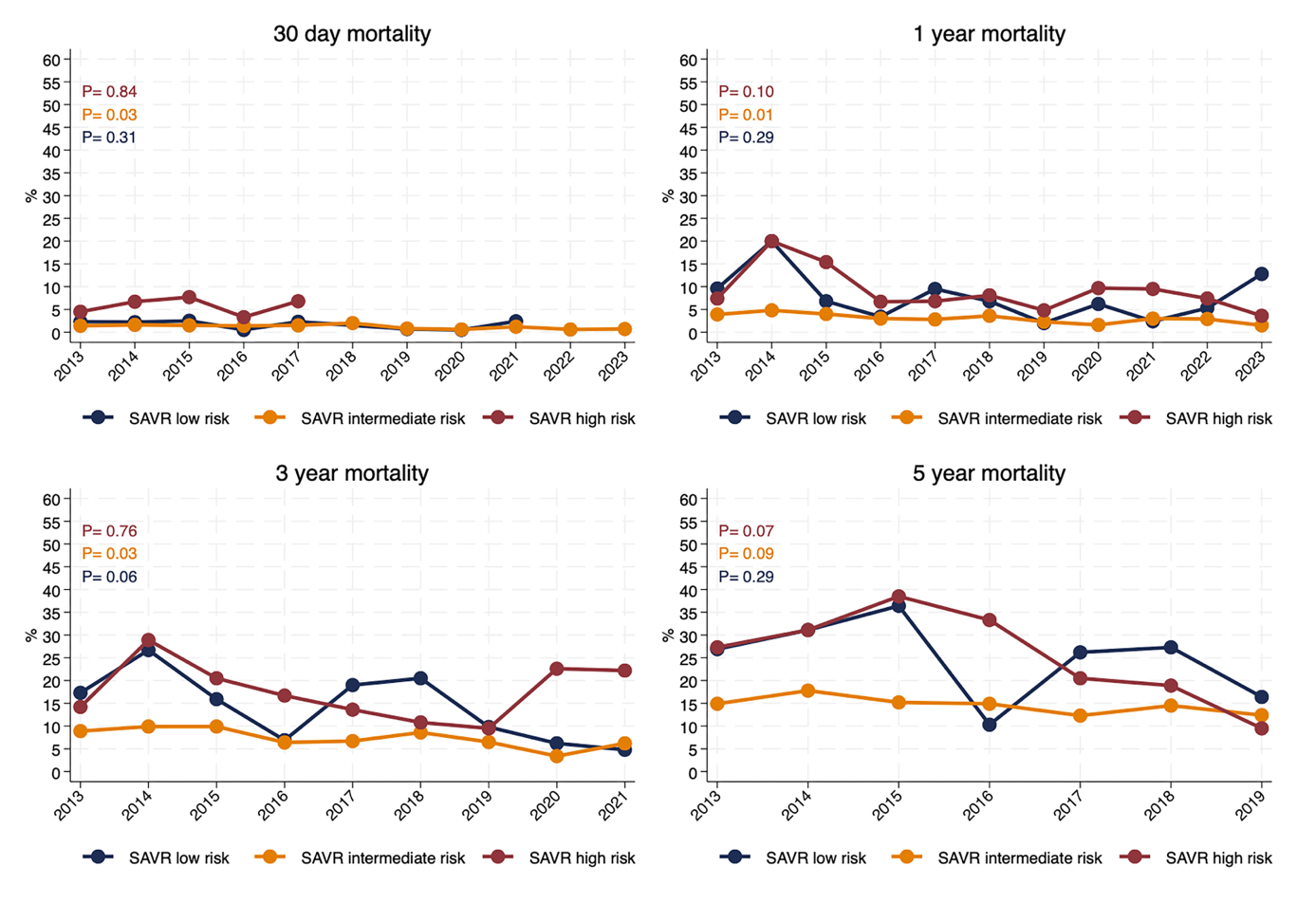


**Supplementary Figure 6. Temporal Trends in All-Cause Mortality at 30days, One, Three, and Five Years for Different Risk Stratifcations of SAVR Patients (2013–2023**)

(a) 30-day all-cause mortality rates over time for SAVR with low-, intermediate and high periprocedural risk (b) One-year all-cause mortality rates over time SAVR with low-, intermediate and high periprocedural risk (c) Three-year all-cause mortality rates over time for SAVR with low-, intermediate and high periprocedural risk (d) Five-year all-cause mortality rates over time for SAVR with low-, intermediate and high periprocedural risk. For SAVR high risk patients in the later years (2018-2023) no estimated events could be calculated due to missing data in the specific subgroup analyses for 30-day mortality. Consistent low mortality was seen across all categories of risk for SAVR patients with no improvement during the study period except for intermediate risk patients in one and three-year mortality.


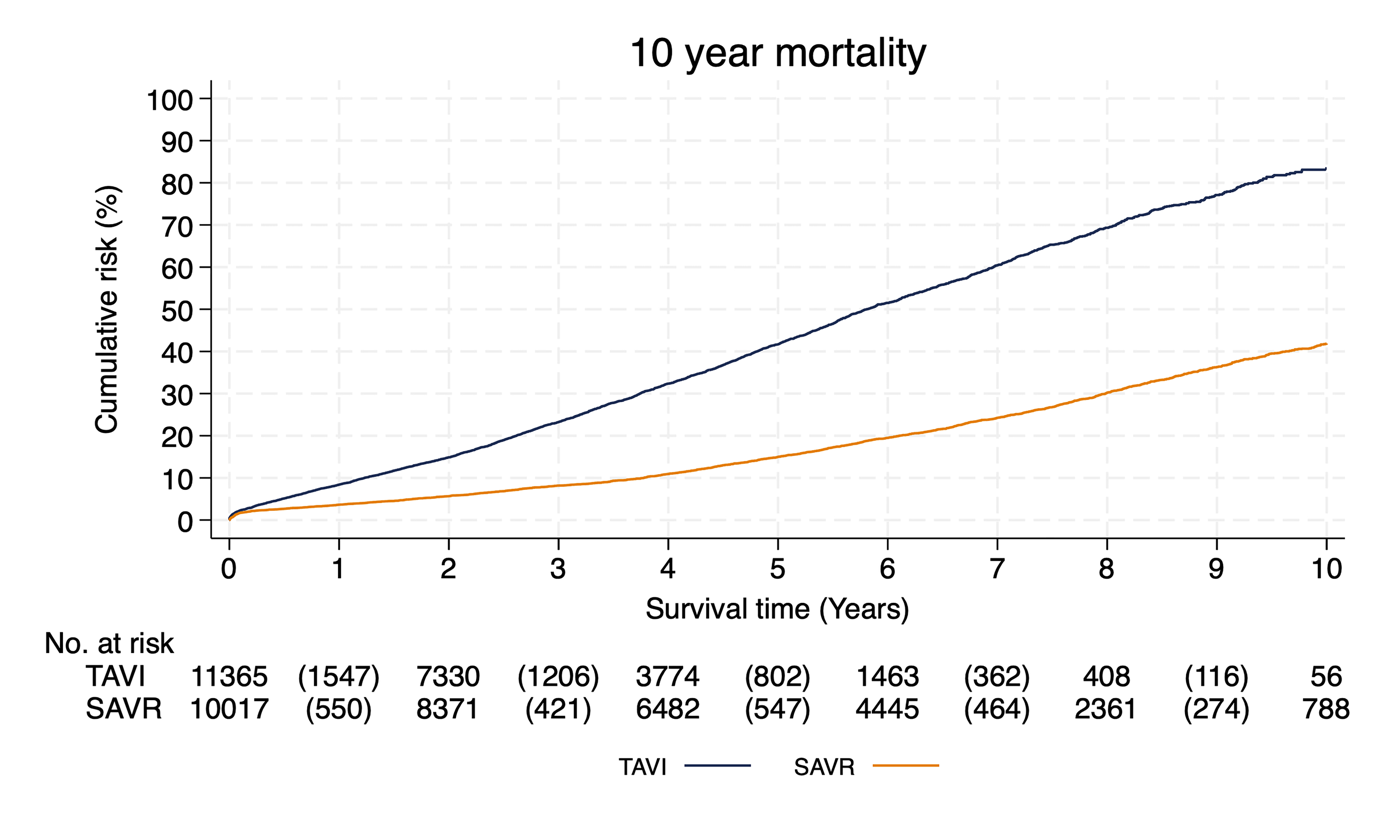


**Supplementary Figure 7. Cumulative Risk of 10 Year All-Cause Mortality for TAVI and SAVR.**

Kaplan–Meier estimates are presented for patients undergoing TAVI and SAVR, including 11,365 TAVI and 10,017 SAVR patients at baseline. Over time, loss to follow-up and censoring substantially reduced the number of patients at risk, leaving only 56 TAVI and 788 SAVR patients in the analysis at 10 years. The unadjusted event rates indicate high long-term mortality among TAVI patients. However, these patients were on average 83 years old at the time of the procedure, and those with available long-term follow-up predominantly represent earlier treatment cohorts with higher baseline surgical risk.

**
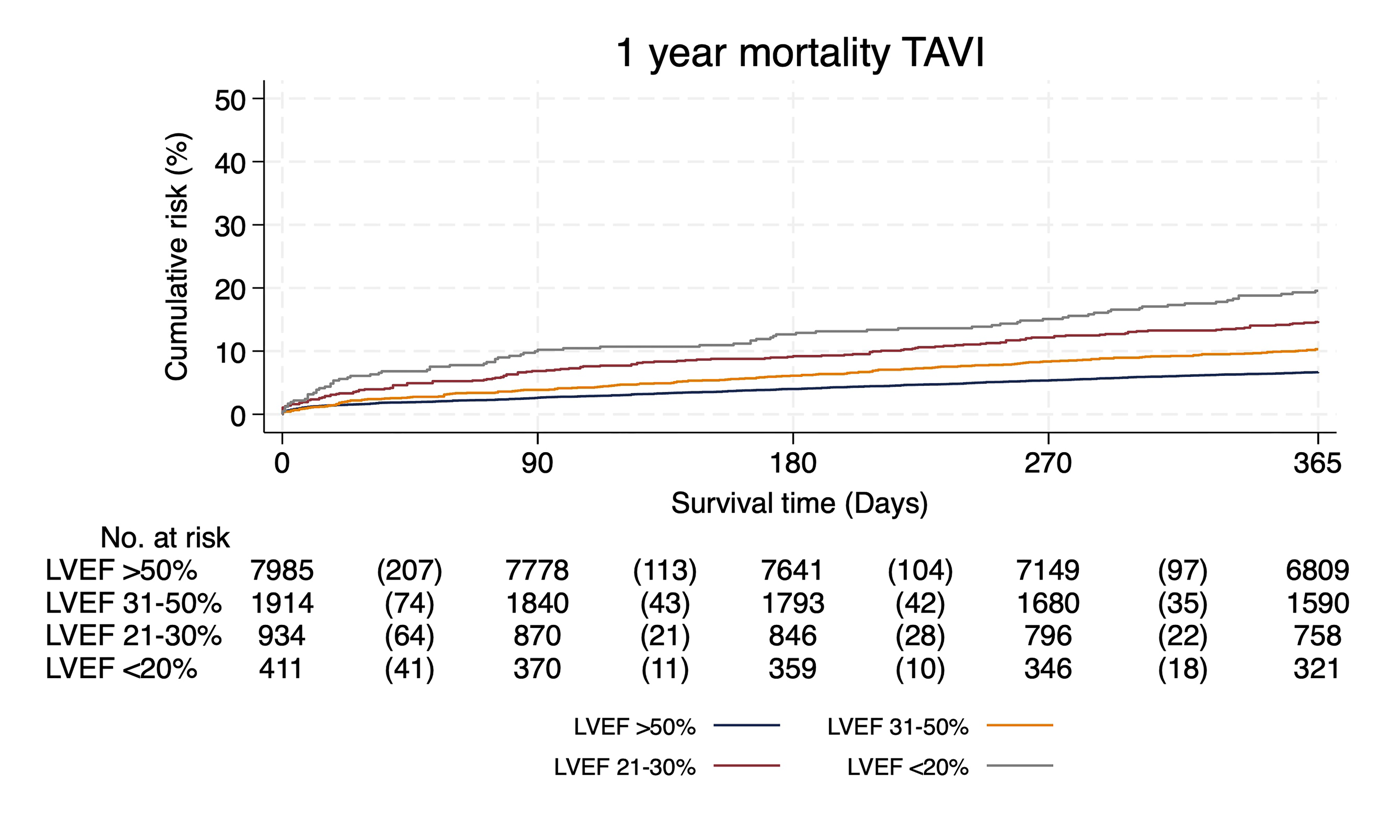
**

**Supplementary Figure 8. Cumulative Risk of 1 Year All-Cause Mortality Rates for TAVI Stratified by Left Ventricular Ejection Fraction.**

Crude cumulative risk of 1 year mortality after TAVI stratified by LVEF. As LVEF decreased the cumulative risk for 1 year mortality increased for each stratified cohort. Largest discriminative risk was seen between normal LVEF and severe reduced LVEF.


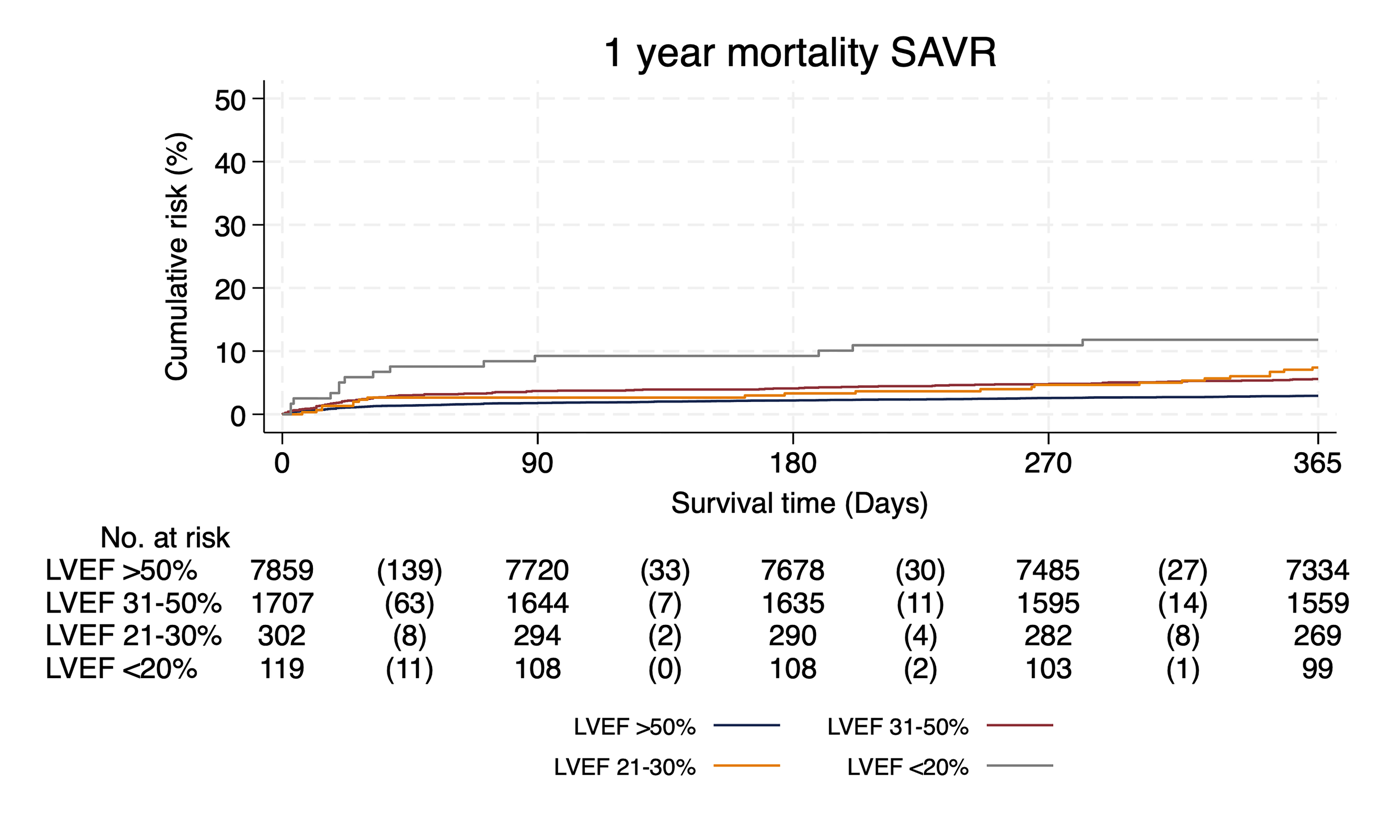


**Supplementary Figure 9. Cumulative Risk of 1 Year All-Cause Mortality Rates for SAVR Stratified by Left Ventricular Ejection Fraction.**

Crude cumulative risk of 1 year mortality after SAVR stratified by LVEF. As LVEF decreased the cumulative risk for 1 year mortality increased for each stratified cohort. Largest discriminative risk was seen between normal LVEF and severe reduced LVEF.


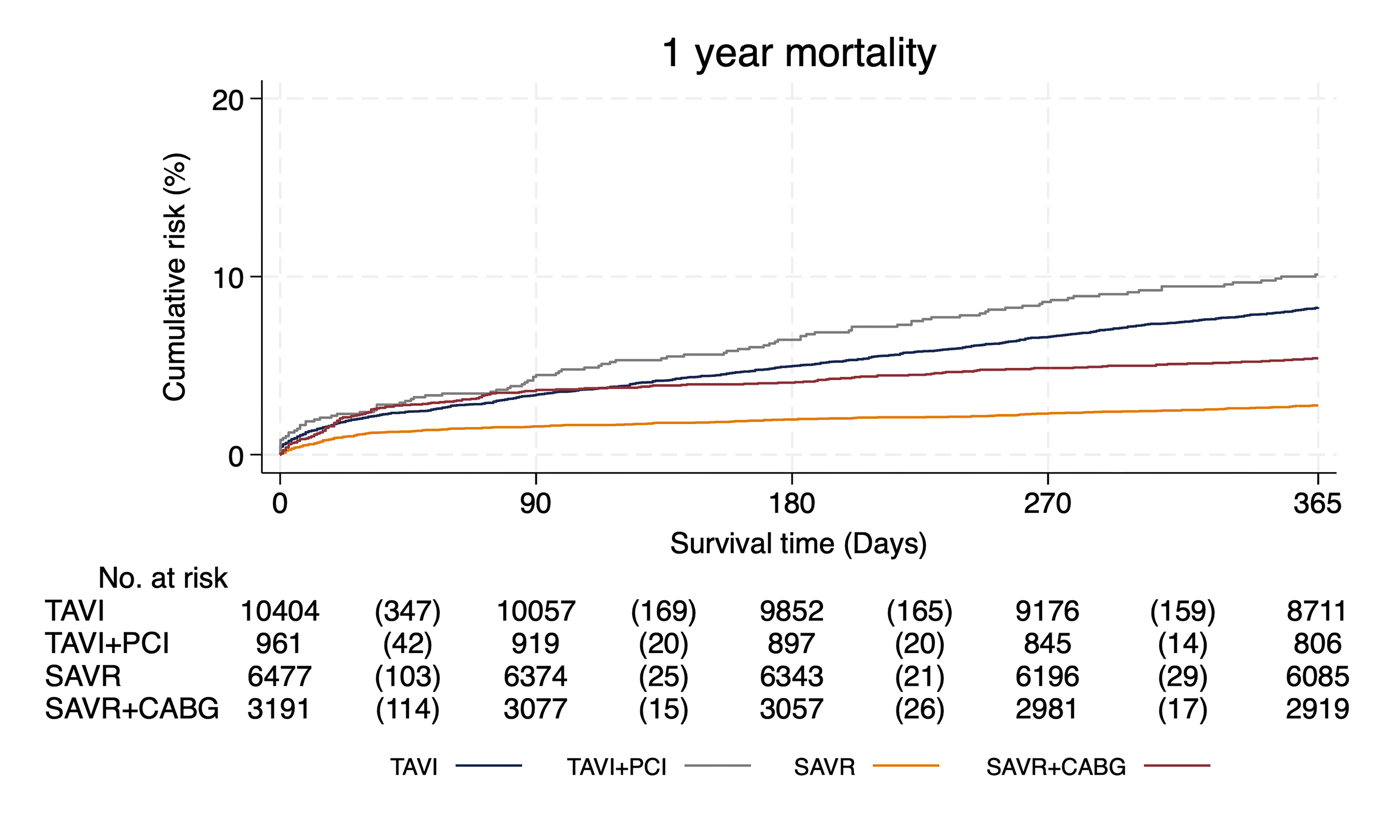


**Supplementary Figure 10. Cumulative Risk of 1 Year All-Cause Mortality Rates for Prosthetic Valve Intervention Stratified by Percutaneous and Surgical as well as Concomitant Coronary Revascularization.**

Crude cumulative mortality rates stratified by intervention modality and concomitant coronary revascularization. Isolated SAVR showed the lowest cumulative risk at 1 year. For SAVR+CABG, the curves diverged from TAVI during the first 3 months but subsequently demonstrated a lower cumulative risk. TAVI+PCI consistently showed the highest 1-year mortality risk compared with the other three modalities.
